# Ultrasensitive nanoscale optomechanical electrometer using photonic crystal cavities

**DOI:** 10.1515/nanoph-2021-0820

**Published:** 2022-03-21

**Authors:** Ji Xia, Qifeng Qiao, Haoyang Sun, Yongjun Huang, Fook Siong Chau, Guangya Zhou

**Affiliations:** University of Electronic Science and Technology of China, No. 2006, Xiyuan Ave, Chengdu, 611731, China; Department of Mechanical Engineering, National University of Singapore, 9 Engineering Drive 1, Singapore, 117575, Singapore

**Keywords:** cavity optomechanics, electrometer, electrostatic spring softening, photonic crystal nanobeam resonator

## Abstract

High-precision detection of electric charge is critical for physical, chemical, and biological measurements. Nanophotonic optomechanical system confines the optical field at the nanoscale and enables a strong interaction between optical cavity and mechanical resonator. Its high optical quality factor cavity and strong optomechanical coupling are promising for precision sensing applications. Here an integrated optomechanical electrometer is proposed for the electric charge sensing using a zipper cavity with a suspended photonic crystal nanobeam (PCN) acting as a movable mechanical resonator. As the electrostatic force arising from the electric voltage to be measured interacts with the mechanical motion of the movable PCN and modulates its resonance through electrostatic stiffening effect, optomechanical coupling transduces the mechanical motion to the optical field with enhanced sensitivity. The resonance shift of the mechanical resonator can be monitored to detect the electric voltage with a sensitivity of 0.007 $\mathrm{Hz}/mV^{2}$ . Moreover, the sensing performance can be further enhanced with the operation of the optomechanical electrometer in the self-sustained oscillation above threshold power. Owing to the narrow-linewidth of detector radio frequency (RF) spectrum with a large peak-to-noise floor ratio (up to 73.5 dB), the enhanced electrical sensitivity of 0.014 $\mathrm{Hz}/mV^{2}$ is achieved with a high resolution of $1.37\;mV^{2}Hz^{-1/2}$ . A theoretical minimal detectable electrostatic charge is calculated as $1.33\times 10^{-2}\;\mathrm{eH}z^{-1/2}$ by converting the measured electric voltage versus RF shift to an approximatively linear relationship. This on-chip optomechanical electrometry scheme provides a powerful solution to the ultrasensitive determination of charged nanoparticles in biological and chemical applications.

## 1 Introduction

Electron is well known as one of the natural elementary particles. Moving of electron flow defines the value of electric voltage and current. For many years, electrometers have been considered as one of the most fundamental instruments for the characterization of electric charge or voltage [1, 2]. Electrometers have found wide applications in the research fields of biological and chemical charge detection [3, 4], environment monitoring [5], mass spectrometry [6, 7], and space exploration [8]. The miniaturized electrometer with high sensitivity is desired for the precise measurement of weak electric voltages, and currents or quantities of electric charges in the research and industry fields. One of the cutting-edge nano-electronic electrometers is conducted using the cryogenically cooled single electron transistor (SET) to achieve a supreme detection resolution, but the SET-based electrometers suffer from limited bandwidth and cannot be operated at room temperature [9, 10]. To overcome these problems, micro- and nano-electromechanical system (M/NEMS) is another great technology to realize the chip-level electrometer with high performance and low energy consumption [1]. Currently, the M/NEMS-based electrometers have been developed for precision sensing based on three approaches, including modulated stiffness [11, 12], vibrating reed [5, 13] and coupled resonators [14]. Basically, the concept of M/NEMS-based electrometry refers to metrology that measures charge by monitoring the frequency variation of mechanical resonators. M/NEMS resonant sensing scheme has advantages of simple structures, reduced sizes and low power consumptions as well as simple postprocessing circuits. The working mechanisms for these sensitive M/NEMS-based electrometers rely on the electrostatic spring softening (ESS) effect. The voltage (*V*) or charged particle (*Q*) on the micro- or nano-scale capacitor results in an electrostatic force or stain on the movable structures, which could modify the effective stiffness of the mechanical resonator. Subsequently, an electrical circuit is developed to read the frequency shift of the vibration mode $\Delta \Omega_{m}∼V^{2}$ or $Q^{2}$ in the form of voltage or charge [15]. In order to realize a high-resolution and sensitivity of the electrical measurement, in general, M/NEMS electrometer requires a cryogenically cooled operation condition, a high-quality resonator as well as a low electronic noise level of readout circuits [4]. However, limited by the inevitably intrinsic mechanical damping loss, M/NEMS electrometers currently could achieve the highest charge resolution of 0.17 $\mathrm{eH}z^{-1/2}$ [12].

Silicon photonics has attracted much attention over the past two decades [16]. Its CMOS compatibility with the semiconductor industry makes it a cost-effective approach to realize the optical routing at a chip level. Using moveable on-chip optical components by electrostatic force, the M/NEMS-enabled photonic integrated circuits can also serve as a superior approach for electrical detection [17]. In contrast to traditional M/NEMS-based sensing systems using the electric circuits to read out the signal, the optical readout mechanism has less detrimental noise reaching quantum-limited level and immunity to external electromagnetic interference [18]. Optical measurement and control of mechanical motion attract great attention from fundamental researches and industrial applications [19], [20], [21]. In particular, the nanophotonic optomechanical system localizes light to subwavelength volume, simultaneously enhancing optomechanical interaction between the localized optical radiation field and mechanical vibration mode at a nanoscale [20, 22]. In the cavity optomechanical system, the mechanical oscillation with a displacement amplitude (*x*) translates into perturbation of cavity mode and hence shifts the optical cavity resonance (*ω*_c_). At the same time, the mechanical motion modulates the intracavity field with a mechanical frequency $\Omega_{m}$ coupled in the optical readout amplitude, which is proportional to the displacement *x* and the optomechanical coupling strength $g_{\mathrm{OM}}=\partial \omega_{c}/\partial x$ . The motion of the mechanical resonator with an effective mass $m_{\mathrm{eff}}$ in the optomechanical system is closely related to the external force $F_{\text{ex}}$ , represented by the mechanical susceptibility $\chi_{xx}\left(\Omega \right)={\left[m_{\mathrm{eff}}\left(\Omega_{m}^{2}-\Omega^{2}-i\Omega \Gamma_{m}\right)\right]}^{-1}=x\left(\Omega \right)/F_{\mathrm{ex}}\left(\Omega \right)$ , where $\Gamma_{m}$ = $\Omega_{m}/Q_{m}$ is mechanical damping rate. The mechanical susceptibility is significantly enhanced at its mechanical resonance $\Omega_{m}$ with a factor of $Q_{m}$ , which indicates that the motion sensitivity could be highly improved by the mechanical resonance with a high mechanical quality factor $Q_{m}$ (or equivalently, a low mechanical damping rate can be achieved by optomechanical backaction [22], which overcomes the high damping rate existed in M/NEMS resonant electrometers). In light of this optically modified effective damping rate, a chip-scale electrometry could be developed based on a cavity optomechanical system. Meanwhile, the optical readout sensitivity can be greatly enhanced by the optical resonance with an ultrahigh optical quality factor $Q_{o}$ . Thanks to the strong coupling in the optomechanical system, it enables an immense platform for external stimulus-induced motion detection with its sensitivity down to several atto meters per square root of hertz [23]. Based on the ultrasensitive motion measurement [24], [25], [26], precision sensing of various physical factors, including mass sensing [27, 28], force sensing [29, 30], accelerator [31], [32], [33], magnetometry [34, 35], atomic force microscope [36, 37] and acoustic sensing [38, 39], etc., have been realized in the optomechanical systems. Furthermore, optomechanical sensors are also endowed with the advantages of compact size, low power consumption, on-chip integration capability and compatibility with fiber coupling [21]. Implementations of cavity optomechanics using the on-chip optical cavity can greatly enhance the optomechanical interaction [19, 40]. There are typically three optical configurations including Fabry–Perot, whispering gallery mode and photonic crystal (PhC) resonators. Among these optical cavities, PhC cavities are highly preferred due to their high optical quality factors, small mode volumes (ultra-high *Q*_o_/*V*), greatly reduced mass reaching a magnitude of a picogram and strong optomechanical coupling inside the nanocavity. Besides, a one-dimensional (1D) PhC cavity can be integrated with movable structures in a flexible configuration which provides a profound platform for precision measurements [31, 41].

Currently, research efforts on using electrostatic force in optomechanical systems mainly focus on electrostatic actuation to control the interaction of the micro-opto-electro-mechanical-systems (MOEMS) [42]. In most of MOEMS devices, the electrostatic force acts as an actuation for optical re-configuration or mechanical switching which could be promising for a range of photonic applications including frequency conversion between electrical and optical domains [43]. On the other hand, several theoretical optomechanical schemes were analytically proposed for electrical charge measurements, including the sensing schemes utilizing optomechanically induced transparency [44, 45], and the nonlinear optomechanical interaction [46]. However, the related experimental work has rarely been demonstrated for electrical voltage or charge detection so far. In this paper, a nanoscale optomechanical electrometer is proposed and experimentally demonstrated for electric voltage detection. The key component of optomechanical electrometer is a zipper cavity that consists of two coupled one-dimensional photonic crystal nanobeam (PCN) cavities where one of the suspended PCN cavities works as a movable mechanical resonator. Perturbed by an electrostatic field on the motion of the movable mechanical resonator, the optomechanical cavity provides a sensitive approach for the precision probing of the weak electric voltage by tracking the RF signal from the cavity readout. Especially, when the pump optical power exceeds the intrinsic mechanical damping loss (above threshold power), the mechanical motion turns into a self-sustained optomechanical oscillation, yielding an optomechanical backaction limited RF linewidth with an amplified oscillation amplitude. Compared to the electric measurement operating below the threshold, the self-sustained optomechanical system is distinguished with a larger sensitivity of 0.014 $\mathrm{Hz}/mV^{2}$ and a minimized resolution of $1.37\;mV^{2}Hz^{-1/2}$ .

## 2 Optomechanical electrometer design and fabrication

**Figure 1:**
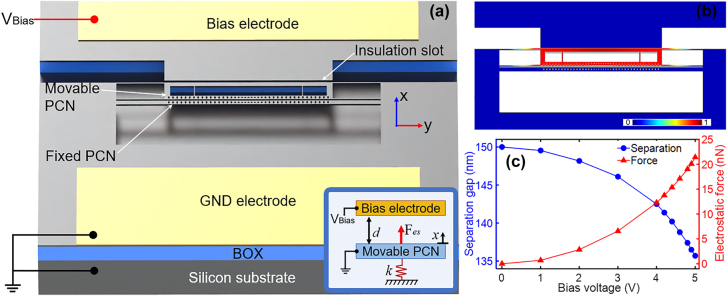
Overview of the nanoscale optomechanical electrometer. (a) Schematic diagram of the electrometry integrated with an optomechanical zipper cavity. Inset: Schematic of the optomechanical electrometry based on a parallel-plate capacitor. (b) The normalized FEM deflection of movable PCN forced by an electric potential. (c) Simulation results of the nanoscale capacitor under the applied bias voltage, the blue data point is the lateral separation of the isolation gap while the red data point shows the electrostatic force induced by the applied voltage.

As shown in Figure 1, the proposed chip-scale optomechanical electrometer is operated to measure the bias voltage (*V*_bias_) by an electro-opto-mechanical interaction from a zipper cavity. As seen from Figure 1(a), the nanophotonic zipper cavity consists of two PCN cavities. One of the PCN cavities is suspended with four flexural beams acting as a mechanical resonator (namely movable PCN). The optomechanical coupling occurs when the other fixed PCN cavity is radiation-driven by the pump light. This zipper cavity and driving bias electrode are electrically isolated by an air slot with an initial separation gap *d* = 150 nm. Besides, the silicon device layer patterned with the zipper cavity together with the silicon substrate layer is electrically grounded to avoid the floating voltages and the undesirable vertical electrostatic force on the zipper cavity. The sidewalls of the movable PCN and the stationary bias electrode form a nanoscale parallel-plate capacitor, which gives rise to an electrostatic force as presented in the inset of Figure 1(a). As shown in Figure 1(b), with an electric potential applied to this capacitor, the isolation slot gap is decreased due to the deflection of movable PCN driven by the electrostatic force. It can also be seen from FEM analysis in Figure 1(b) and(c) that the air isolation slot reduces while the electrostatic force quadratically increases as a function of the bias voltage. The electrostatically driven motion of the movable PCN can shift the optical resonance of the zipper cavity and modulate the natural frequency of movable mechanics. Subsequently, the optomechanical dynamical-backaction behaviours of the zipper cavity are determined under the combined actions of optical force and electrostatic force. Therefore, the precise measurement of the electrical voltage can be realized by observing the RF readout of the movable PCN.

**Figure 2:**
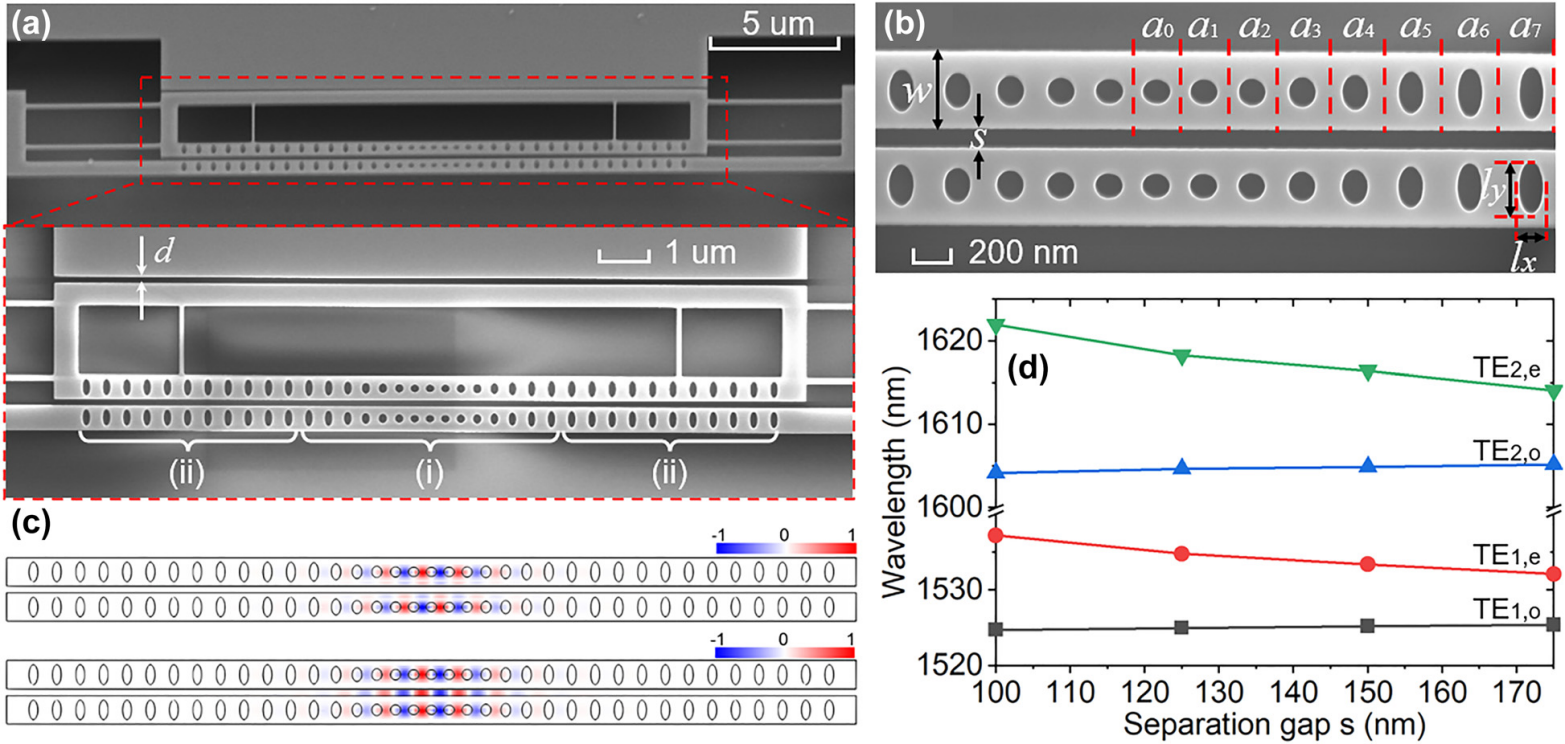
Optomechanical zipper cavity. (a) SEM images of the nanoscale optomechanical electrometer on an SOI chip. Inset: Zoom-in view of the zipper cavity electrically isolated with a bias electrode with a gap width *d*. The PCN cavity is formed with defect region (i) and mirror region (ii). (b) SEM image of the zipper PCN cavities that are parameterized with beam width *w* and beam slot *s*. $a_{i}\left(i=0,1,2,\ldots ,7\right)$ is the lattice constant of unit cell in the defect region. $l_{x}$ and $l_{y}$ are major and minor axes of the elliptical air holes in the mirror region. (c) FEM simulated in-plane electrical field of the fundamental optical cavity modes $TE_{1,o}$ (top) and $TE_{1,e}$ (bottom). (d) Simulated resonance wavelength shift of zipper cavity under an increasing gap width.

As illustrated in Figure 2(a), the zipper cavity is formed with a pair of coupled PCN resonators with a slot gap. The fixed PCN cavity is used for light pumping by a dimpled fiber taper due to its larger rigidity. The other PCN cavity is suspended by four 120 nm-wide and 5 μm-long silicon beams. As a result, the entire suspended structure enables a feasible in-plane mechanical movement under the combined actuation of optical gradient force and electrostatic force. The nanoscale PCN cavity is fabricated from the 220 nm thick silicon device layer that is patterned with (i) a parameterized transition of air hole and unit cell in the defect region (ii) and two symmetric mirror regions. To achieve high optical quality resonant modes, the physical dimensions of PCN cavity (Figure 2(b)) are designed with reference to the developed optimization methods [41, 47] by Lumerical FDTD simulations. In this work, the PCN cavity is featured with 15 holes in the central defect region and 11 mirror holes at two sides of the nanobeam to achieve desirable cavity mode frequencies and high optical *Q*-factors. The PCN cavity is specified by the unit cell geometric sizes of the defect region varying gradually from the defect unit values (*a*_0_, *w*, $l_{x}$ , $l_{y}$ ) = (329, 505, 200, 174) nm to the nominal unit values (*a*_7_, *w*, $l_{x}$ , $l_{y}$ ) = (435, 505, 168, 370) nm. When two single PCN cavities are placed in the near-field of each other with a separation slot gap, the resulting strong mode coupling between these two identical PCN cavities leads to TE-polarized even and odd parity supermodes. As presented in Figure 2(c), the fundamental resonant even mode (TE_1,e_) has much more electric field localized inside the slot gap than that of the odd mode (TE_1,o_). FEM results indicate that the nanophotonic zipper with a slot gap *s* = 125 nm employed here supports cavity TE_1,o_ mode at the wavelength of 1525.0 nm with a $Q_{o}=2.6\times 10^{7}$ and TE_1,e_ mode at the wavelength of 1534.8 nm with a $Q_{o}=3.2\times 10^{7}$ . Moreover, the optical resonances of this zipper cavity are highly dependent on the variation of slot gap (plotted in Figure 2(d)). The simulated results in Figure 2(d) indicate that the resonant wavelengths of the fundamental and second order even modes are shifted to the shorter wavelengths and those of the odd modes move towards the longer wavelengths as the slot gap (*s*) increases. Meanwhile, it can also be seen that the resonant even modes are endowed with a larger wavelength shift at the same variation of slot gap. Specifically, the simulated results in Figure 2(d) show that the optomechanical coupling strength of TE_1,e_ mode is calculated as $g_{\mathrm{OM}}/2\pi \approx 15\;\mathrm{GHz}/\mathrm{nm}$ , which is much larger than that of TE_1,o_ mode $g_{\mathrm{OM}}/2\pi \approx 1.92\;\mathrm{GHz}/\mathrm{nm}$ . Therefore, we primarily focus on the fundamental even mode in this optomechanical cavity for this work because of its larger optomechanical coupling strength. With the resonant mode field being largely confined within the slot gap between two PCNs, the resonances of the zipper cavity are sensitively coupled to the in-plane motion of the movable PCN (the x direction in Figure 1(a)). Hence, a displacement of movable PCN induced by the electric bias voltage applied to the on-chip integrated nanoscale capacitor can be optically read out using the optomechanical system. The proposed nanoscale optomechanical electrometer is developed on silicon-on-insulator (SOI) wafter from SOITEC (resistivity 8.5–11.5 ohm-cm, buried-oxide (BOX) layer thickness 2 µm). The fabrication process is CMOS-compatible (see Supplementary I). Firstly, the electrometer patterns, including the optical cavity, movable structure and isolation trenches, are defined on the silicon device layer by electron beam lithography and a following deep reactive ion etching of silicon. Secondly, the metal electrode pattern is achieved by thermal deposition and a lift-off process. Finally, the movable features are enabled by the removal of the BOX layer by the vapor HF release. For the measurement of electric voltage, the gold pads on device are wire bonded to a printed circuit board to achieve electrical connections.

## 3 Measurements and analysis

### 3.1 Experimental setup and optical characterization of optomechanical electrometer

**Figure 3:**
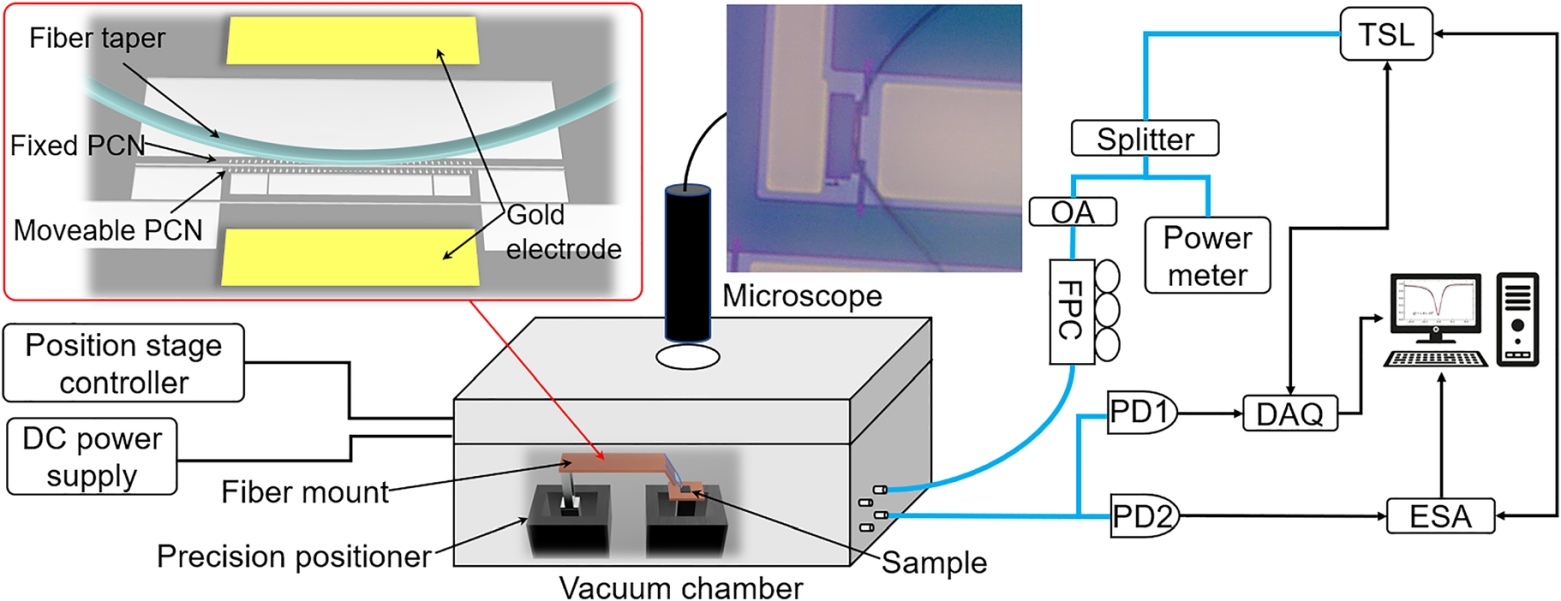
Experimental setup for the optomechanical electrometer. Inset indicates the precise alignment of fiber taper and on-chip zipper cavity. A microscopic image shows the observation of fiber taper attached to the fixed PCN cavity.

Characterization of the optomechanical electrometer is performed under a low-pressure environment (1×10^−3^ mbar) and at room temperature. As shown in Figure 3, light launching from a tunable laser (TSL, Santec TSL710, Japan) is split into two parts: 90% optical intensity into subsequent optical fiber circuits and 10% intensity to a power meter to monitor the light stability. The 90% pump light is sent to an optical attenuator to reduce the pump light intensity when necessary to avoid optical nonlinear effects. Then its polarization is adjusted to excite transverse electric (TE) mode through a fiber polarized controller to ensure an efficient optical coupling between the dimpled fiber taper and the zipper cavity. Next, this TE-polarized light is launched into the on-chip device. Light escaping from the zipper cavity is coupled back into the fiber via the same fiber taper and is directly received by two photodetectors (PDs). Subsequently, signal from PD1 (10 MHz bandwidth, Femto FMG-OE-300-IN-01-FC, Germany) is sent to the data acquisition device (DAQ) and signal from PD2 (125 MHz bandwidth, Newport 1811, USA) is sent to the electrical signal analyzer (ESA, Agilent N9020A, USA). To characterize the optical properties of the optomechanical electrometer, synchronization is established between DAQ and TSL to obtain the swept optical spectrum from PD1. Meanwhile, the PD2 signal sent to ESA is monitored to obtain the mechanical properties of the optomechanical electrometer. In the detection of mechanical spectra, the laser wavelength should be set and swept across the optical resonance by simultaneously controlling ESA and TSL to obtain the RF spectra as a function of laser-cavity detuning. Both optical and mechanical spectra data are finally processed in the computer. For purpose of detection of electric voltage, a DC power supply (Agilent E3631A, USA) is used to generate and calibrate the electric potential between the zipper cavity and bias electrode. In this work, a dimpled fiber taper is evanescently coupled with the zipper cavity, as illustrated in the inset of Figure 3. The fiber taper is precisely positioned with a triaxial nanopositioner, and with the help of an optical microscope, the dimple of fiber taper is accurately aligned and mechanically attached with the rigid side of the zipper cavity (i.e. fixed PCN).

**Figure 4:**
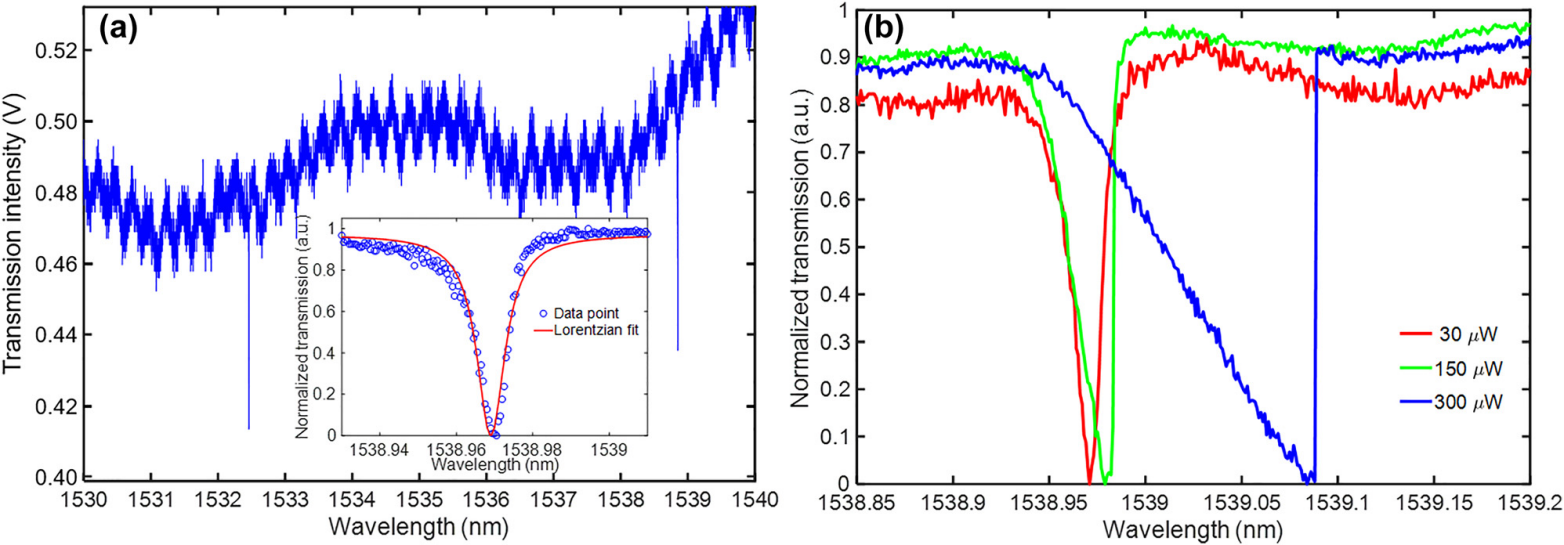
Optical transmission spectrum of the optomechanical zipper cavity. (a) The fundamental TE_1,o_ and TE_1,e_ modes of the zipper cavity. Inset shows the resonance of TE_1,e_ mode with Lorentzian fitting to obtain an optical *Q*-factor up to $1.5\times 10^{5}$ . (b) Resonance broadening of TE_1,e_ modes with the increasing pump power.

First, we investigate the optical properties of the optomechanical electrometer. The optical transmission spectrum through the fiber taper is used to observe the optical resonance of zipper cavity. When the cavity is driven by a pump laser intensity of 30 μW in the telecom band (1480–1630 nm), the fundamental and the second-order modes of cavity can be observed in the transmission spectrum. As shown in Figure 4(a), the fundamental modes TE_1,o_ and TE_1,e_ of the zipper cavity are measured at *λ*_1,o_ = 1532.469 nm and *λ*_1,e_ = 1538.892 nm, respectively. With a Lorentzian fitting of the TE_1,e_ resonance, full width at half maximum of 10 pm (*Q*_o_ ≈1.5×10^5^) can be found. These measured results are in good agreement with the FEM results which indicates a high-quality fabrication of device. With the increase of pump laser power, as presented in Figure 4(b), the line shape of the optical resonance becomes increasingly asymmetrical and broadened due to the thermal and optomechanical nonlinearities inside the zipper cavity.

**Figure 5:**
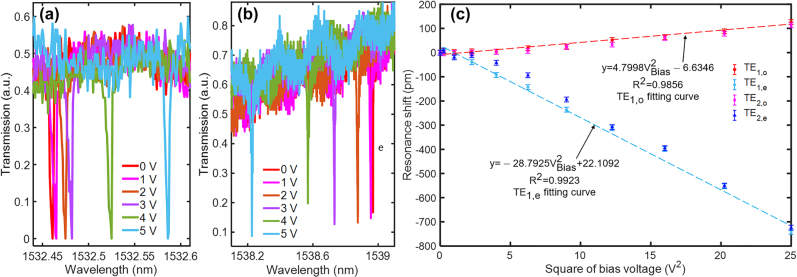
Electrostatic force-induced optical resonance shift of the zipper cavity. (a) and (b) optical resonant wavelength shift of TE_1,o_ and TE_1,e_ under the increasing applied bias voltages. (c) Resonance shift of the fundamental and second-order optical modes under the increasing bias voltages (from 0 V to 5 V, step 0.5 V) with a linear fitting to identify the dispersion sensitivity.

To verify the electrostatic force-induced resonance shift of the zipper cavity, the optomechanical electrometer is driven by an increasing bias voltage from 0 V to 5 V. Figure 5(a) and (b) indicate the resonant wavelength shifts of the detected modes of the zipper cavity. With an applied bias voltage on the optomechanical electrometer, an electrostatic force $F_{\mathrm{es}}=\frac{\varepsilon tLV_{\mathrm{Bias}}^{2}}{2d^{2}}$ ( ε is vacuum dielectric constant; *t* = 220 nm, *L* = 16.32 μm are the thickness of nanobeam and the length of movable PCN, respectively) is acted on the movable PCN to push it towards the bias electrode in the *x*-axis direction. The movement of PCN leads to a decreasing isolation gap *d* but an increasing slot gap *s* that affects the optomechanical coupling strength of the zipper cavity. It can be seen that a red resonant frequency shift in the TE_1,o_ mode (Figure 5(a)) and a blue resonant frequency shift in the TE_1,e_ mode (Figure 5(b)) are observed with the applied voltage increasing from 0 V to 5 V (corresponding to a simulated $F_{\mathrm{es}}$ reaching up to roughly 21.4 nN). The mechanical deflection of the movable PCN shifts the optical resonance, and this relationship can be evaluated as $\left|\Delta \lambda \right|=\left|\alpha V_{\mathrm{Bias}}^{2}\right|$ with an optical tunability of the electrometer α to identify its sensitivity. It can be found that the maximum resonance shift of TE_1,e_ mode (742 pm) is much larger than that of TE_1,o_ mode (126 pm) due to the higher optomechanical coupling strength of even mode resonances. As summarized in Figure 5(c), there is a good linear relationship between the resonance shift and the applied square of bias voltage, which agrees well with the FEM simulation and theoretical calculations. The sensitivity of TE_1,e_ resonance is calculated as |*α*_1,e_|= 28.8 pm/*V*^2^, and that of TE_1,o_ resonance is $\left|\alpha_{1,o}\right|=$ 4.8 pm/*V*^2^. Considering the high *Q*_o_ of TE_1,e_ mode and its larger sensitivity, the minimum detectable electric voltage using the optical resonance shift is determined as ${\left(\delta V_{\min}\right)}^{2}=\frac{\lambda_{1,e}}{\left|\alpha_{1,e}\right|}\frac{1}{1-T_{d}}\frac{1}{Q_{o}}\frac{1}{\mathrm{SNR}}\approx 0.026V^{2}$ (where $T_{d}$ is the dip ratio of transmission spectrum at the resonance, and SNR is the ratio of off-resonance power to noise level. See Supplementary II).

### 3.2 Characterization of optomechanical electrometer below threshold power

**Figure 6:**
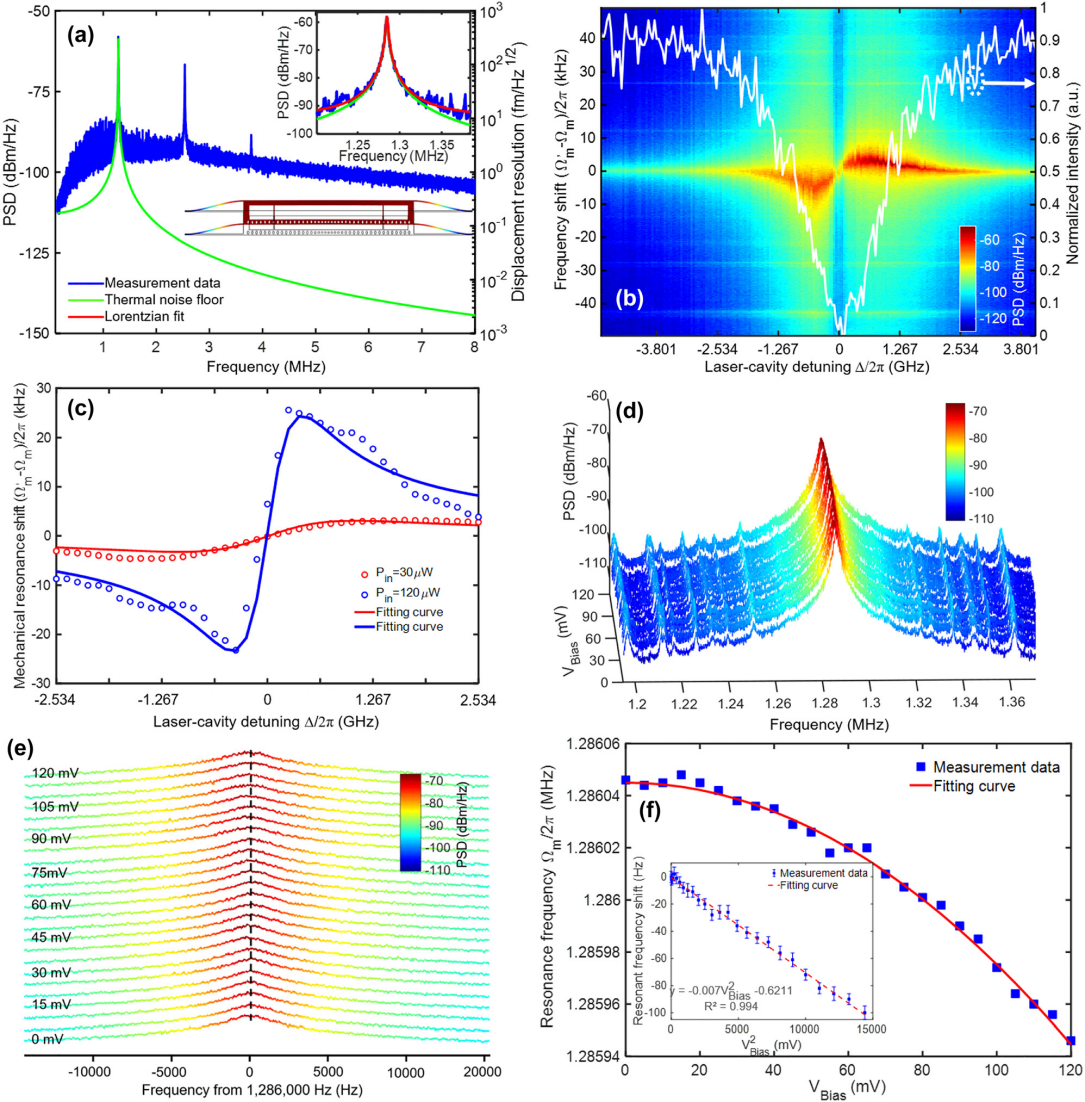
RF spectra of the optomechanical electrometer below the threshold power. (a) Measured RF spectrum of the fundamental in-plane mechanical mode of the moveable PCN (resolution bandwidth (RBW) of ESA is set at 100 Hz). Insets are FEM-calculated in-plane mechanical mode of the movable PCN and zoom-in view of the RF spectrum of the fundamental vibrating mode at $\Omega_{m}/2\pi =1.286\;\mathrm{MHz}$ , respectively. The blue-colored curve presents the measured data, the green-colored curve is the theoretically calculated thermal noise floor, and the red-colored curve is the Lorentzian fitting curve for the mechanical resonant peak. (b) PSD map of the measured RF spectrum as functions of laser-cavity detuning and mechanical resonance shift from the in-plane fundamental mode to verify the optical spring effect of the optomechanical electrometer. At the pump power of 30 μW, the RF spectrum is obtained by sweeping through the TE_1,e_ resonance as plotted with the white-colored curve. (c) Mechanical resonance shift versus laser-cavity detuning for different pump powers. The discrete symbols denote the Lorentzian-fitted peak frequencies from the measured data (15 μW for red data points and 120 μW for blue data points), and the red and the blue curves are the numerical modeled fitting curves. (d) RF transduction at the pump power 30 μW for measurement of applied voltages from 0 mV to 120 mV. (e) Zoom-in view of the RF peaks of the spectra under applied voltages. The black dashed curve indicates the variation of RF peaks as a function of the applied bias voltage. (f) Mechanical resonant frequency versus applied voltages. Inset is the measured data converted to a linear relationship between the resonant frequency shift and applied voltage.

The in-plane mechanical displacement of movable PCN in the optomechanical electrometer is sensitively coupled to the optical resonance of the zipper cavity due to a strong optomechanical interaction. With the laser wavelength detuned at the shoulder of the optical resonance, this in-plane motion is optically read out by ESA. When the optomechanical cavity is pumped with an incident power of *P*_in_ = 30 μW, the in-plane fundamental mechanical mode and other two harmonic peaks of the movable PCN arise from its thermal Brownian motion, as shown in the power spectral density (PSD) spectrum in Figure 6(a). The FEM simulated in-plane eigenmode profile of movable PCN in the inset agrees well with the measured frequency $\Omega_{m}/2\pi =1.286\;\mathrm{MHz}$ and the effective mass of the movable PCN can be extracted as $m_{\mathrm{eff}}=k/\Omega_{m}^{2}=22.6\;\mathrm{pg}$ with a calculated spring constant of *k* = 1.47 N/m. A Lorentzian fitting of the mode peak data in the experimental spectrum indicates that the mechanical quality of the eigenmode reaches to $Q_{m}\approx 1280$ under a low pressure. The right axis of Figure 6(a) describes the optical transduced displacement PSD spectra from the power PSD spectra (see Supplementary III). In the unresolved sideband (*Ω*_m_≪*κ*, *κ*=*ω*_c_/*Q*_o_) with laser-cavity detuning set at Δ=κ/2 , the displacement of movable PCN coupled to the optical intensity transmission and transduced to the frequency component of optical power is given by $P_{xx}=\left(1-T_{d}\right)\frac{Q_{o}}{\omega_{c}}g_{\mathrm{OM}}P_{\det}x$ , where $P_{\det}$ is the optical power received by the detector off the resonance. It shows that the in-plane eigenmode in power PSD unit is equivalently transduced to the displacement noise unit, reaching a measured displacement noise floor of $∼0.8\;\mathrm{fm}/\sqrt{\mathrm{Hz}}$ . The green solid line depicts the theoretically calculated thermal noise level background of this eigenmode (see Supplementary IV). In this nanoscale optomechanical cavity, the optomechanical backaction [22] induced by the optical force not only allows for precision measurement of in-plane motion but also strongly modifies the rigidity of the nanomechanical resonator and RF spectra of the output. By monitoring the mechanical spectra under the laser-cavity detuning values without electrostatic perturbation, optical spring effect is observed. As shown in Figure 6(b), a spring stiffening occurs in the blue-detuned regime and a spring softening occurs in the red-detuned regime, respectively. At a low pump power *P*_in_ = 30 μW, an example optical TE_1,e_ resonance around 1538.35 nm is plotted in the white-colored line with an estimated optical intracavity energy of 50 aJ, which corresponds to the cavity photon number of *n*_c_≈ 410. The mechanical resonant frequency shift over a large detuning range can be verified by the effective mechanical frequency $\Omega_{m}^{\prime }$ relation [19, 22] in the unresolved sideband,

(1) $$\Omega_{m}^{\prime }=\sqrt{\Omega_{m}^{2}+\left(\frac{2{\left|\hat{a}\right|}^{2}g_{\mathrm{OM}}^{2}}{\Delta_{\text{o}}^{\text{′}2}\omega_{c}m_{\mathrm{eff}}}\right)\Delta }$$

where, $\Delta =\omega_{l}-\omega_{c}$ is the laser-cavity detuning and $\Delta_{o}^{\prime 2}=\Delta^{2}+{\left(\kappa /2\right)}^{2}$ . Figure 6(c) extracts the mechanical frequency shift over a laser detuning range of 40 pm under the pump power of 15 μW and 120 μW, respectively. As shown in Figure 6(c), the peak and valley of the mechanical resonance shift under 15 μW are loaded at the laser-cavity detuning of Δ/2π≈±0.63 GHz  (equals to a laser−cavity detuned wavelength of∓5 pm) and the largest mechanical resonant frequency shift is $\left(\Omega_{m}^{\prime }-\Omega_{m}\right)/2\pi \approx 3.5\;\mathrm{kHz}$ . Meanwhile, fitting the mechanical resonant frequency shift versus laser-detuning using Eq. (1) can also be used to evaluate the optomechanical coupling strength as $g_{\mathrm{OM}}/2\pi \approx 13.2\;\mathrm{GHz}/\mathrm{nm}$ , which is slightly lower than the FEM simulated value due to the imperfect fabrication of the released structures. Increasing the pump power to 120 μW yields a stronger optical force which enlarges mechanical resonant frequency shift up to $\left(\Omega_{m}^{\prime }-\Omega_{m}\right)/2\pi \approx 26\;\mathrm{kHz}$ . Therefore, the increasing intracavity energy could result in a larger slope of mechanical resonant frequency shift, which in turn may enable an enhancement of detection sensitivity.

With the laser wavelength and power fixed (pump laser wavelength at 1538.345 nm and pump power 30 μW), the optomechanical electrometer can measure the applied bias voltages by monitoring the cavity readout and detecting the mechanical resonance shift. It should be noted that the energy stored in the parallel-plate capacitor charged with *V*_Bias_ = 120 mV is calculated as about 1.5 aJ , which is much smaller compared to the optical intracavity energy. With the applied bias voltage, the resulting nonlinear electrostatic force exerts feedback to modify the mechanical stiffness (namely, ESS effect) of the nanomechanical resonator, which is similar to the optical spring effect in the cavity optomechanics [48]. Here, under the applied bias voltage on the nanoscale parallel-plate capacitor, the mechanical resonant frequency shift in a linearized ESS region (when $\left|k_{\mathrm{es}}|\ll |k_{0}\right|$ ) is given by,

(2) $$\delta \Omega_{m}=\Omega_{m}-\sqrt{\frac{k_{0}+k_{\mathrm{es}}}{m_{\mathrm{eff}}}}=\Omega_{m}\frac{\varepsilon tLV_{\mathrm{Bias}}^{2}}{2d^{3}k_{0}}=\beta V_{\mathrm{Bias}}^{2},\;\;\left(k_{\mathrm{es}}<0\right)$$

where $k_{0}$ is the mechanical spring constant of the selected in-plane mode of the movable PCN and $k_{\mathrm{es}}$ is the negative mechanical stiffness subjected to the ESS effect, and β is defined as the mechanical tunability to quantify the sensitivity. Eq. (2) indicates that the mechanical resonant frequency shift of the optomechanical cavity has a linear relationship with the square of applied voltage. In our experiments, an increasing voltage from zero to 120 mV with a step voltage of 5 mV is applied on the bias electrode, and the resulting mechanical resonant frequency shift as a function of the applied voltage is presented in Figure 6(d). A zoom-in view in Figure 6(e) is captured from the mechanical resonance peak region in Figure 6(d) to show a slight resonance shift of about 100 Hz at a bias voltage of 120 mV. Using Lorentzian fitting to extract the peak position of the RF spectrum, Figure 6(f) further shows the relationship between the mechanical resonant frequency and the applied bias voltage, which is consistent with Eq. (2). The test result shown in Figure 6(f) indicates an experimental sensitivity of β= 0.007 Hz mV^−2^ (or a scale factor of *S*_1_=*β*^−1^= 142.8 mV^2^ Hz^−1^).

### 3.3 Measurement of optomechanical electrometer above threshold power

**Figure 7:**
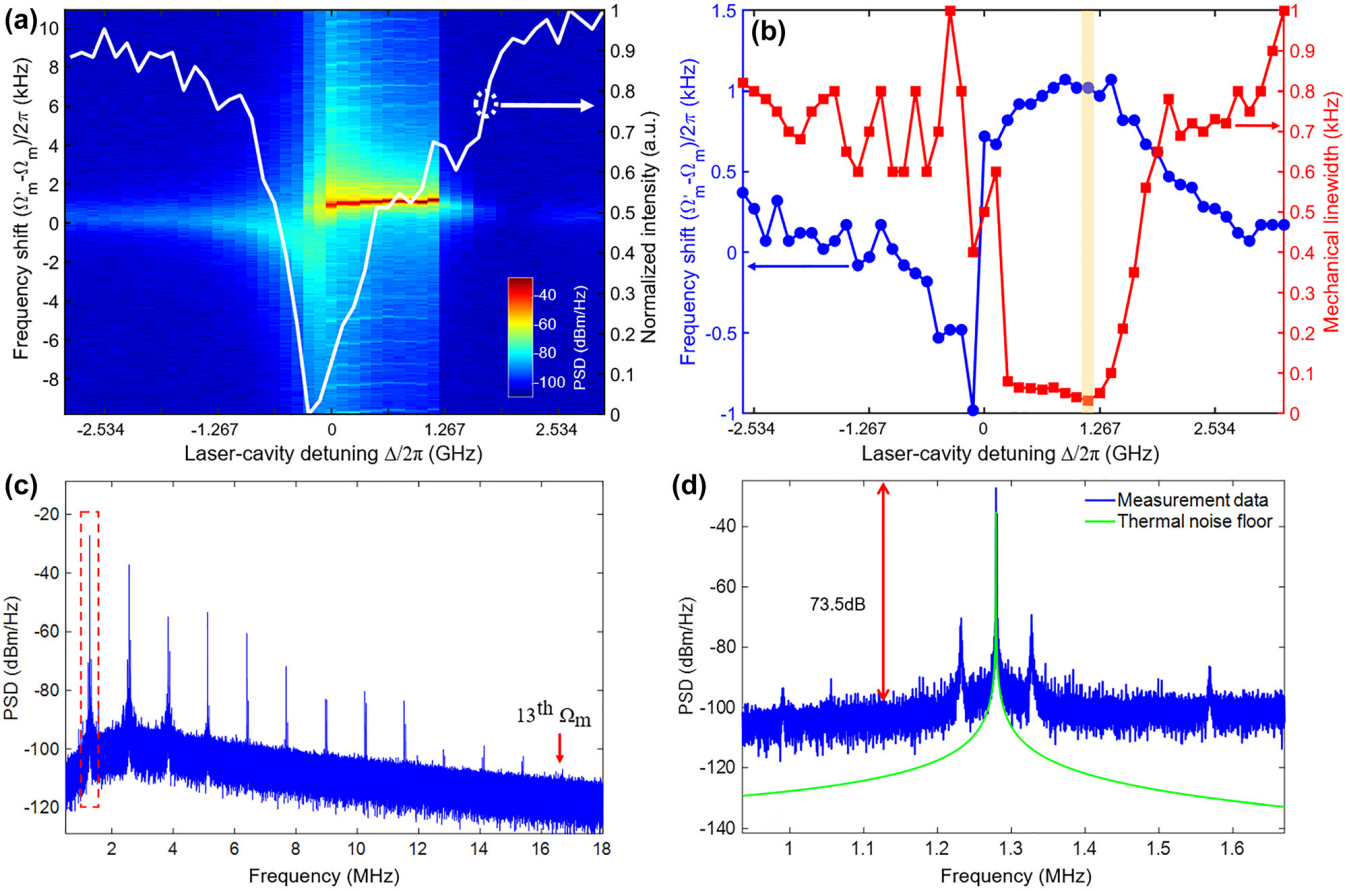
Optomechanical backaction RF spectra above threshold power. (a) PSD map of the measured RF spectrum as functions of laser-cavity detuning to verify and mechanical resonance shift from the in-plane fundamental mode above the threshold power. At the pump power of 180 μW, the RF spectrum is obtained by sweeping through the cavity TE_1,e_ resonance as the white-colored curve. (b) Mechanical resonant frequency shift and mechanical linewidth versus laser-cavity detuning at the pump power of 180 μW. The light-yellow region indicates the operation position of the pump light for bias voltage detection. (c) RF harmonics of the optomechanical cavity above the threshold, up to the 13th harmonic at 16.7 MHz. The RBW is 100 Hz. (d) Zoom-in view of the spectrum in the red-dashed box of Figure 7(c). The RBW is 1 Hz to characterize the RF spectrum. The green-colored curve is the theoretically calculated thermal noise floor.

When the optomechanical electrometer works above the threshold power, a self-sustained optomechanical oscillation is realized with a narrow mechanical linewidth and a high oscillation amplitude (see Supplementary V). As shown in Figure 7(a), tuning the optomechanical cavity through the optical TE_1,e_ resonance (white-colored curve) with a detuning range of 50 pm with a pump power *P*_in_ = 180 μW (above threshold), the RF-spectra indicates a significant parametric oscillation effect in the blue-detuned regime. Due to the nonlinear optical resonance resulting from the high pump power, as presented in Figure 7(b), both the optical spring and damping effects have only emerged in the blue-detuned regime. At the laser-cavity detuned wavelength of 8 pm (equals to the blue-detuned frequency of about 1 GHz), a self-induced optomechanical oscillation reaches a minimal linewidth of *Γ*_m_/2*π*≈ 12 Hz (corresponding to an effective mechanical *Q*_m_ ≈ 1×10^5^). Here, the mechanical resonance linewidth is greatly reduced compared with that of the optomechanical cavity driven below threshold power. It is noted that this optomechanical electrometer operating in the self-sustained oscillation mode can greatly reduce the mechanical damping rate, thus overcoming the extensive damping rates existed in the traditional M/NEMS resonant electrometers. It is also interesting to note that, due to the increased nonlinearity in the optomechanical nanocavity, high-order harmonics (up to 13th harmonic) of the in-plane fundamental oscillation mode are excited as presented in Figure 7(c). Furthermore, for the fundamental in-plane oscillation mode at $\Omega_{m}/2\pi =$ 1.286 MHz, the peak-to-noise level is significantly enhanced and reaches approximately 73.5 dB as compared to that about 30 dB in the oscillation mode below the threshold power. Besides, two sidebands resulting from the coupling to out-of-plane modes are also observable as shown in Figure 7(d).

**Figure 8:**
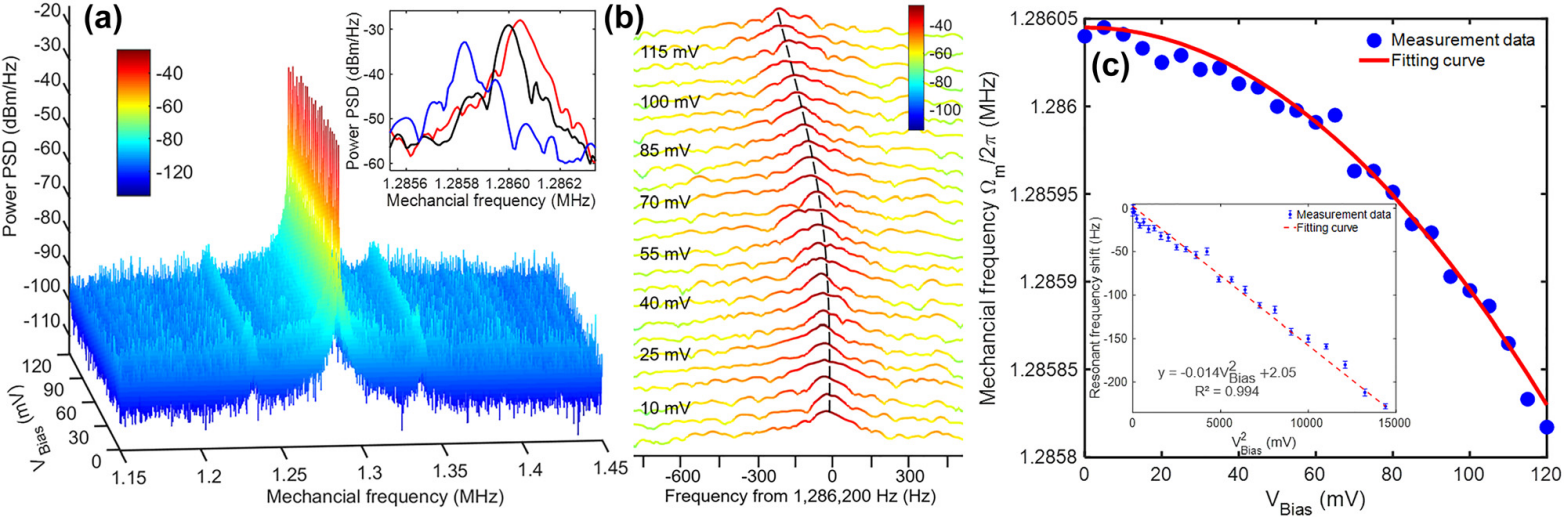
Bias voltage detection in the optomechanical electrometer above threshold power. (a) RF spectra above threshold power tracked for measurement of the applied bias voltage from 0 mV to 120 mV. Inset is the zoom-in view of the resonance peak area to observe the shift of mechanical frequency at 0 mV (red), 60 mV (black) and 120 mV (blue). (b) Zoom-in view of the spectra at the resonance peak area as a function of the applied bias voltages. The black dashed curve indicates the variation of the resonance peaks. (c) Mechanical resonant frequency versus applied bias voltages. Inset is the measured data converted to a linear relationship between resonance shift and applied voltage squared.

Benefiting from the linewidth-narrowing of the mechanical sensing mode, the optomechanical electrometer is driven above the threshold power to track the variation of mechanical resonance frequency shift under various applied bias voltage as shown in Figure 8. Figure 8(a) shows the mechanical resonance spectra of the fundamental in-plane oscillation mode ((in an RBW of 1 Hz) as a function of increasing applied electrical voltage from 0 mV to 120 mV. The inset in Figure 8(a) illustrates three selected peaks of the mechanical resonance spectra under 0 mV (red), 60 mV (black) and 120 mV (blue) to clearly show the ESS effect. Figure 8(b) further highlights a zoom-in view captured from the resonance peak region. Compared to Figure 6(e), a more prominent decreasing resonance shift is observed in Figure 8(b) with a larger frequency shift of 230 Hz due to the ESS effect. Figure 8(c) shows that the extracted peak position of the RF spectra has a quadratic declining trend with the increase of the applied bias voltage, which also agrees well with Eq. (2). Converting the horizontal axis to the square of the applied bias voltage as shown in the inset of Figure 8(c), a linear relation can be fitted with an experimental sensitivity of β= 0.014 Hz/mV^2^ (or a scale factor of $S_{2}=\beta^{-1}=$ 71.4 mV^2^ Hz^−1^). Compared with the measured sensitivity below the threshold, this optomechanical electrometer operating above the threshold has achieved a doubled sensitivity due to the minimal distinguishable mechanical resonance shift.

**Figure 9:**
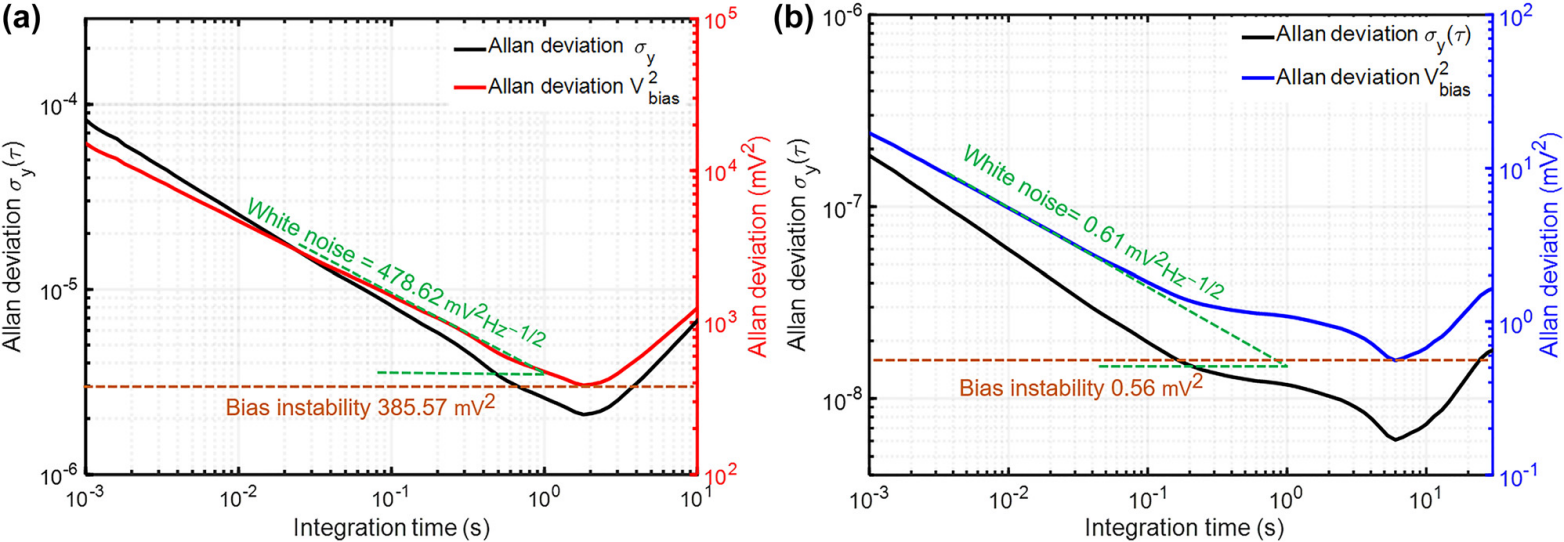
Allan deviation $\sigma_{y}$ as a function of integration time *τ* for the optomechanical electrometer operating below and above threshold power without bias voltage applied, respectively. (a) Allan deviation below the threshold (*P*_in_ = 30 μW), where the black line is resonant frequency Allan deviation and the red line is converted Allan deviation (=*S*×*σ*_*y*_(*τ*)×*Ω*_m_/2*π*) in the term of the square of bias voltage. (b) Allan deviation above the threshold (*P*_in_ = 180 μW), where the black line is resonant frequency Allan deviation and the blue line is the converted Allan deviation in the term of the square of bias voltage. Overall, the bias instability (brown dotted line) and white noise (green dotted line) are estimated at the −1/2 slope and zero slopes.

Moreover, the RF frequency is measured as a function of time to calculate the Allan deviation to estimate the long-term stability of mechanical resonant frequency, as shown in Figure 9. This is also a significant method to evaluate the performance of the optomechanical electrometer as Allan deviation allows extraction of the detection resolution by investigating the noise and stability from the time-domain data. In the analysis on Allan deviation, of greatest importance for sensing applications are white noise and bias instability. The white noise dominants in the lower integration time and it is highly relevant to thermal effects. Besides, the bias instability indicates the minimum achievable resolution limited by flicker noise contribution and fluctuation on the motion of movable PCN or the optical field. When the optomechanical electrometer works below the threshold, as illustrated in Figure 9(a), the Allan deviation of $\sigma_{y}=2.1\times 10^{-6}$ at the zero slope (bias stability) is measured for mechanical resonant frequency $\Omega_{m}/2\pi =1.286\;\mathrm{MHz}$ at 2 s integration. Thus, the measured root-mean-square (RMS) frequency variation in the averaging time of 2 s is identified as $\delta \Omega_{\mathrm{rms}}/2\pi =2.7\;\mathrm{Hz}$ , correspondingly the minimal detectable electric voltage is determined as $R=\left(S_{1}\times \Omega_{m}/2\pi \times \sigma_{y}\right)\sqrt{t_{\mathrm{inter}}}=545.38\;mV^{2}Hz^{-1/2}$ for this optomechanical electrometer. According to the theoretical thermal noise dominated frequency fluctuation extracted as $\delta \Omega_{th}={\left[\left(k_{B}T/E_{C}\right)\times \left(\Omega_{m}\Delta f/Q_{m}\right)\right]}^{1/2}$ from the measured PSD spectrum [11] (where, $k_{B}$ , $E_{C}$ and Δf are Boltzmann constant, carrier energy inside the oscillator and acquisition rate defined by the integration time), a theoretical RMS frequency variation $\delta \Omega_{\mathrm{th}}/2\pi \approx 1\;\mathrm{Hz}$ in the 2 s integration time is calculated based on the measured peak-to-noise level of $10\;\log\left(E_{C}/k_{B}T\right)\approx 30\;\mathrm{dB}$ and $Q_{m}\approx 1280$ . Therefore, converting to the form of a square of electric voltage by $S\times \sigma_{y}\left(\tau \right)\times \Omega_{m}/2\pi$ (red line), the electrical bias instability is 385.57 mV^2^ with a white noise obtained at the −1/2 slope of 478.62 $mV^{2}Hz^{-1/2}$ . As this optomechanical electrometer turns to operate in the self-sustained oscillation status above threshold power, the Allan deviation calculated in Figure 9(b) indicates that bias instability is routinely obtained as $\sigma_{y}=6.1\times 10^{-9}$ for mechanical resonant frequency $\Omega_{m}/2\pi =1.286\;\mathrm{MHz}$ at 6 s integration. Correspondingly, we obtain that the measured RMS frequency variation of 7.8 ×10^−3^ Hz approaches closely to the theoretical thermal noise limited frequency fluctuation of 2.6 ×10^−3^ Hz in the integration time of 6 s. Besides, the converted electric voltage Allan deviation shows that electric bias instability has been greatly improved to 0.56 mV^2^ with a white noise of 0.62 mV^2^ Hz^−1/2^ at the −1/2 slope. Benefiting from the large peak-to-noise floor ratio and ultra-narrow mechanical linewidth in the self-sustained state, the optomechanical electrometer allows a minimal distinguishable mechanical resonance shift which is much smaller than that obtained below threshold power. As a result, the electric resolution of optomechanical electrometer operating above the threshold power is greatly enhanced to $R=\left(S_{2}\times \Omega_{m}/2\pi \times \sigma_{y}\right)\sqrt{t_{\mathrm{inter}}}=1.37\;mV^{2}Hz^{-1/2}$ . According to the thermal Brownian noise dominated detection resolution, the ultimate resolution of the optomechanical electrometer can be solved as $V_{\mathrm{Bias},\mathrm{th}}^{2}=\sqrt{\frac{16k_{B}T\Omega_{m}m_{\mathrm{eff}}d^{4}}{{\left(\varepsilon tL\right)}^{2}Q_{m}}}$ = 0.7 mV^2^ Hz^−1/2^ at ambient temperature, implying that this optomechanical electrometer can reach the high-precision electrical sensing towards the thermodynamical limits. Distinguishing from the detectable resolution of $0.026\;V^{2}$ as the wavelength shift with electric voltage, here the RF readout resolution of the optomechanical measurement raises to $1.37\;mV^{2}Hz^{-1/2}$ with a significant improvement rate of $∼10^{5}$ (approximately equals to the effective *Q*_m_). It demonstrates that the minimal detectable variation of RF readout of optomechanical electrometer is greatly enhanced with a rate dependent on both optical and mechanical quality factors. In particular, when the optomechanical electrometer works in the electrically prebiased regions [12] (for example, offset bias voltage *V*_bias_≥ 70 mV), the quadratic relation between the mechanical resonant frequency shift and applied bias voltage can be transformed into an approximately linear sensitivity. In the linearized region, this optomechanical electrometer achieves a sensitivity of 3 Hz/mV with a corresponding resolution of $R_{\mathrm{linear}}\approx 6.34\times 10^{-3}\;\mathrm{mV}Hz^{-1/2}$ . Besides, simplifying the nanoscale parallel-plate capacitor consisting of two sidewalls of the movable PCN and stationary bias electrode on the silicon device layer, a small capacitance is calculated as C=εtL/d=0.21 fF . Therefore, this proposed optomechanical nanocavity based electrometry could theoretically achieve a charge resolution of $1.33\times 10^{-2}\mathrm{eH}z^{-1/2}$ . Compared to the stare-of-the-art M/NEMS resonator-based electrometers [4, 12], the proposed optomechanical electrometer exhibits an extremely significant improvement in the resolution for electrical measurements with the measurement noise close to the thermal noise limit.

## 4 Conclusions

In this paper, a nanoscale optomechanical electrometer is experimentally demonstrated using the self-sustained oscillation in cavity optomechanics, for the first time, to enhance the resolution of electric voltage detection. Owing to the strong optomechanical coupling in a zipper cavity, the optical readout of mechanical resonant frequency shift of a movable PCN resonator enables the sensitive detection of bias voltage. Compared to traditional M/NEMS resonant electrometers, this optomechanical electrometer has the following outstanding advantages and improvements: (1) we implement cavity optomechanics to realize a CMOS-compatible electrometer with low noise and high sensing resolution. This optomechanical electrometer demonstrates a significant sensing scheme for electric charge detection in the MOEMS devices, which is superior to traditional M/NEMS sensors considering its sensing performance (see the performance comparison in Supplementary VI). (2) In this optomechanical electrometer, the light pump excites an optical force-driven interaction between the optical cavity and the movable mechanical resonator, and optical readout of mechanical resonant frequency shift allows probing of electrical voltage. Compared with the electronic noises limited M/NEMS resonant electrometers, this all-optical light pump and readout approach could suppress these noises arising from the electrical readout circuits effectively and allow the detection noise level close to the thermal noise limit. (3) Instead of optical readout of resonant wavelength shift, or linewidth and amplitude variation of optical resonance, this optomechanical electrometer employs the RF readout to characterize the performance of the sensor. Benefiting from the strong optomechanical coupling and the dynamic backaction induced amplification (e.g., the self-sustained oscillation), a combination of large $g_{\mathrm{OM}}$ , high optical $Q_{o}$ as well as ultra-narrow $\Gamma_{m}$ (equivalently, high effective mechanical $Q_{m}$ ) contributes to a significantly improved resolution of the sensor. (4) Optomechanical electrometer has distinctive characteristics of controllable dynamical backactions, such as laser-detuned optical spring and amplification, allowing for the improved sensing performance. Optical spring effect dependent on the laser-detuning could modify the effective stiffness of mechanics for increased sensitivity. The amplified mechanical oscillation above the threshold could result in a greatly improved resolution. (5) Optimization of the functional mechanical oscillator in the optomechanical electrometer could further enhance the detection resolution. According to the theoretical thermodynamics limited resolution of the optomechanical electrometer, optimization on the device geometry and mechanical stiffness can further enhance the performance of the proposed sensor. For example, reducing the operating frequency of the nanomechanical oscillator can effectively improve the sensing resolution by utilizing a soften support beam Furthermore, using advanced nanofabrication technology, a smaller separation gap could be developed in the optomechanical electrometer, allowing for more precise electrical charge measurement. In addition, introducing a large mass oscillator could also reduce the thermal Brownian noise effectively and enable a lower frequency instability, resulting in a better sensing resolution.

To summarize, we have proposed and investigated an optomechanical electrometer for the first time in this work. When the optomechanical electrometer works below threshold power, an electrical sensitivity of 0.007 Hz/mV^2^ is obtained with an inadequate resolution of $545.38\;mV^{2}Hz^{-1/2}$ limited by the fluctuation of RF frequency and low $Q_{m}$ . Moreover, as the optomechanical electrometer is driven above the threshold power, it turns into the self-sustained oscillation state with an ultra-narrow mechanical linewidth and a significantly increased peak-to-noise floor ratio. In this self-sustained oscillation, the proposed electrometer shows a sensitivity of 0.014 Hz/mV^2^ with an extremely enhanced resolution of $1.37\;mV^{2}Hz^{-1/2}$ . Converting the electric voltage to charge quantification, this electrometer could achieve a theoretical charge probing resolution approaching the single-electron level of $1.33\times 10^{-2}\;\mathrm{eH}z^{-1/2}.$ Assisted by the optomechanical transduction and RF readout scheme operating at the mesoscopic room temperature, this electrometer could potentially provide a low-noise and high-precision measurement platform beyond single electron for the next generation of electrical instruments.

## Supplementary Material

Supplementary Material
