# Supplementary material for: Ultrasensitive nanoscale optomechanical electrometer using photonic crystal cavities
Source: Nanophotonics. 2022 Mar 21;11(8):1629–42. doi: 10.1515/nanoph-2021-0820 (PMC11502092; doi:10.1515/nanoph-2021-0820)
Supplement: Supplementary file 1 — Supplementary Material [file j_nanoph-2021-0820_suppl.docx]

Supplementary Information

**Ultrasensitive Nanoscale Optomechanical Electrometer using Photonic Crystal Cavities**

Ji Xia^a^, Qifeng Qiao^a^, Haoyang Sun^a^, Yongjun Huang^b,^*, Fook Siong Chau^a^, Guangya Zhou^a,^*

^a^ Department of Mechanical Engineering, Faculty of Engineering, National University of Singapore, Singapore 117575, Singapore

^b^ School of Information and Communication Engineering, University of Electronic Science and Technology of China Chengdu 611731, China

Corresponding authors: Guangya Zhou and Yongjun Huang

*Email: mpezgy@nus.edu.sg (G.Y. Zhou); yongjunh@uestc.edu.cn (Y.J. Huang)

### I. Fabrication of optomechanical electrometer

In this experiment, all electro-optomechanical devices are fabricated on silicon-on-insulator (SOI) wafer with a 0.22 μm thick silicon layer and 2 μm buried oxide (BOX) layer, including the PCN resonators, waveguides and other nanomechanical structures. As shown in Fig. S.1(a), several types of optomechanical electrometers with different mechanical stiffnesses are designed and fabricated on this SOI chip, and two electrometers are electrically isolated by the isolation trenches. The long gold electrodes connected with gold pads on the edge of chip are employed to apply the electric signal to be measured. The device used in our measurements is marked with a blue square in Fig. S.1(b), which consists of the optomechanical zipper cavity and electrodes.


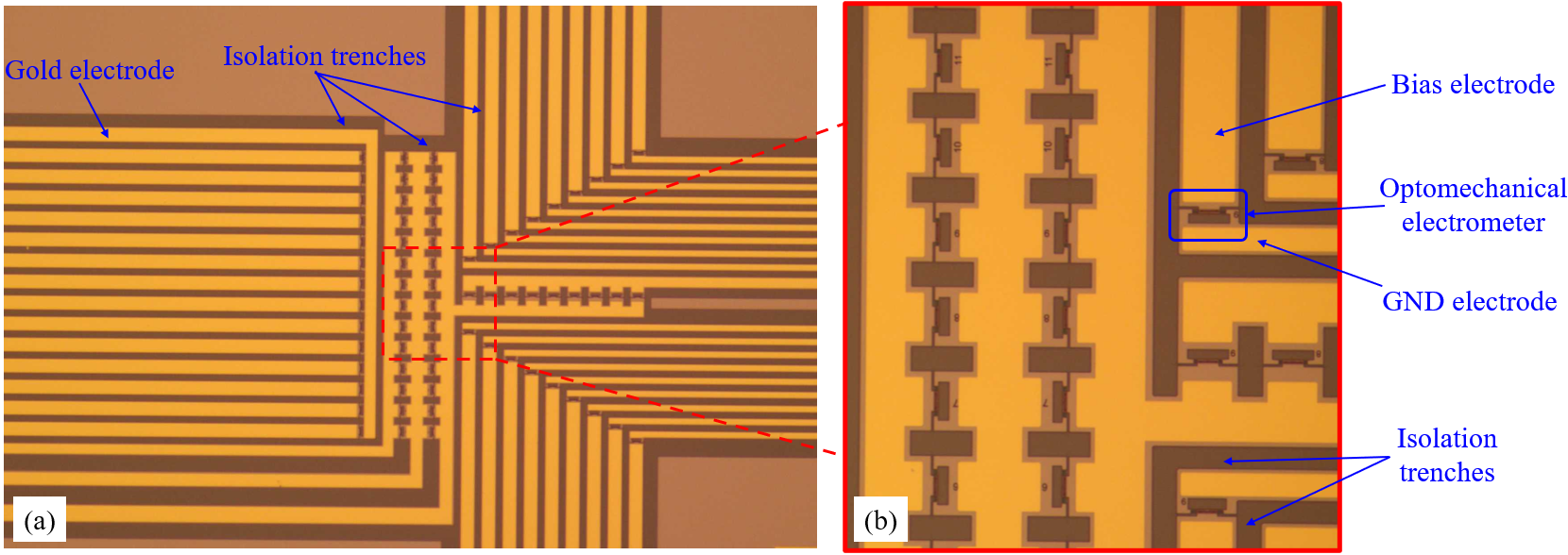


**Figure S.1.** Microscopic image of the optomechanical electrometer devices. (a) On-chip electrometer devices with the gold electrical circuits and the isolation trenches; (b) Optomechanical electrometer tested in the experiments.

Fabrication work of these devices are mostly finished in Tianjin H-chip Technology Group Corporation, including the general operations of electron beam lithography (EBL), deep reactive ion etching (DRIE), laser writer lithography, inductively coupled plasma (ICP) etching, electron beam deposition, thermal deposition, hydrofluoric acid vapor (VHF) release and scanning electron microscope (SEM). The suspended optomechanical electrometer devices are fabricated on a 2cm×2cm SOI chip wafer, which mainly includes five key steps: metal marker deposition, optomechanical device etching, laser writer lithography of isolation trenches, gold electrodes deposition and VHF for suspended structure. The detailed fabrication process is briefly described as shown in Fig. S.2.

The first section is metal marker deposition which includes Steps 1-5. This metal marker is used as alignment reference points outside of the devices for different pattern layers. PMMA photoresist is coated on the surface of silicon layer through a 4500 rpm spin coating (Step 2). Then, an EBL is used to expose the metal marker pattern in 1000μC/cm^2^ (Step 3). A thermal evaporator is used to deposit an adhesion layer and a following gold (Au) layer (Step 4). For the samples that required BOX layer removal, Chromium (Cr) should be used as the adhesion layer to avoid the HF liquid attack. We adopt a 10 nm Cr and 50 nm Au deposition for this alignment marker.

After the alignment marker is finished, EBL and a following DRIE are used to obtain the optomechanical devices in the second section includes Steps 6-9:

- Step 6: EBL resist coating for pattern definition process. A 3500 rpm spin coating is used to coat a layer of 220 nm thickness ZEP 520 beam resist on the surface of the silicon layer. A 280 $\text{μC}\text{/}\text{cm}^{\text{2}}$ dose with a 200 pA current is used for EBL exposure. Subsequently, this cavity pattern has been defined and then transferred to the silicon layer for the silicon etching.
- Step 7: DRIE of silicon device layer. The defined cavity pattern works as an etching mask in this dry etching. Besides, ICP is also used in this etching step to achieve a high aspect ratio and smooth sidewall of the waveguide to reduce the optical loss.
- Step 8: Removal of EBL resist for the next electrical isolation trenches etching.

In the third section (Steps 10-13), etching of electrical isolation trenches for the electro-optomechanical devices. These trenches in a large size could provide an electrical isolation between two neighboring devices and define the top silicon layer area for following gold electrode deposition. After the ZEP 520 photoresist is spin-coated on the surface of the silicon device layer (Step 10), the pattern of isolation trenches is exposed using an EBL. A 360 $\text{μC}\text{/}\text{cm}^{\text{2}}$ dose with a 5 nA current is used for lithography exposure (Step 11). Then, DIRE is used to etch the silicon layer for the trenches (Step 12). Finally, the photoresist is removed for the following gold electrode deposition (Step 13).

The fourth section including Steps 14-17 is to deposit the electrodes. In the same manner, the PMMA photoresist is coated on the surface of the silicon layer through a 4500 rpm spin coating (Step 14). And EBL is used to expose the gold electrode pattern in 1000 μC/cm^2^ (Step 15). Subsequently, a thermal evaporator is used to deposit an adhesion layer (10 nm thick Cr) and the following 50 nm gold layer (Step 16). Finally, the removal of the remaining resist is finished by the lift-off operation (Step 17).

In the final section (Step 18), VHF release is implemented to achieve the suspended structures in the optomechanical electrometer. To achieve the movable structures, the sacrificial BOX layer beneath the silicon device layer is removed by HF vapor. To avoid the release stiction, the dry HF vapor is used in this step. The process is generally carried out by a HF concentration of 99.99% under the pressure of 100 Torr and the temperature of 70 ℃. Typically, the oxide etching rates at vertical and lateral directions are in the 0.1 microns/minute range that is determined by the dimension of cavity structure.


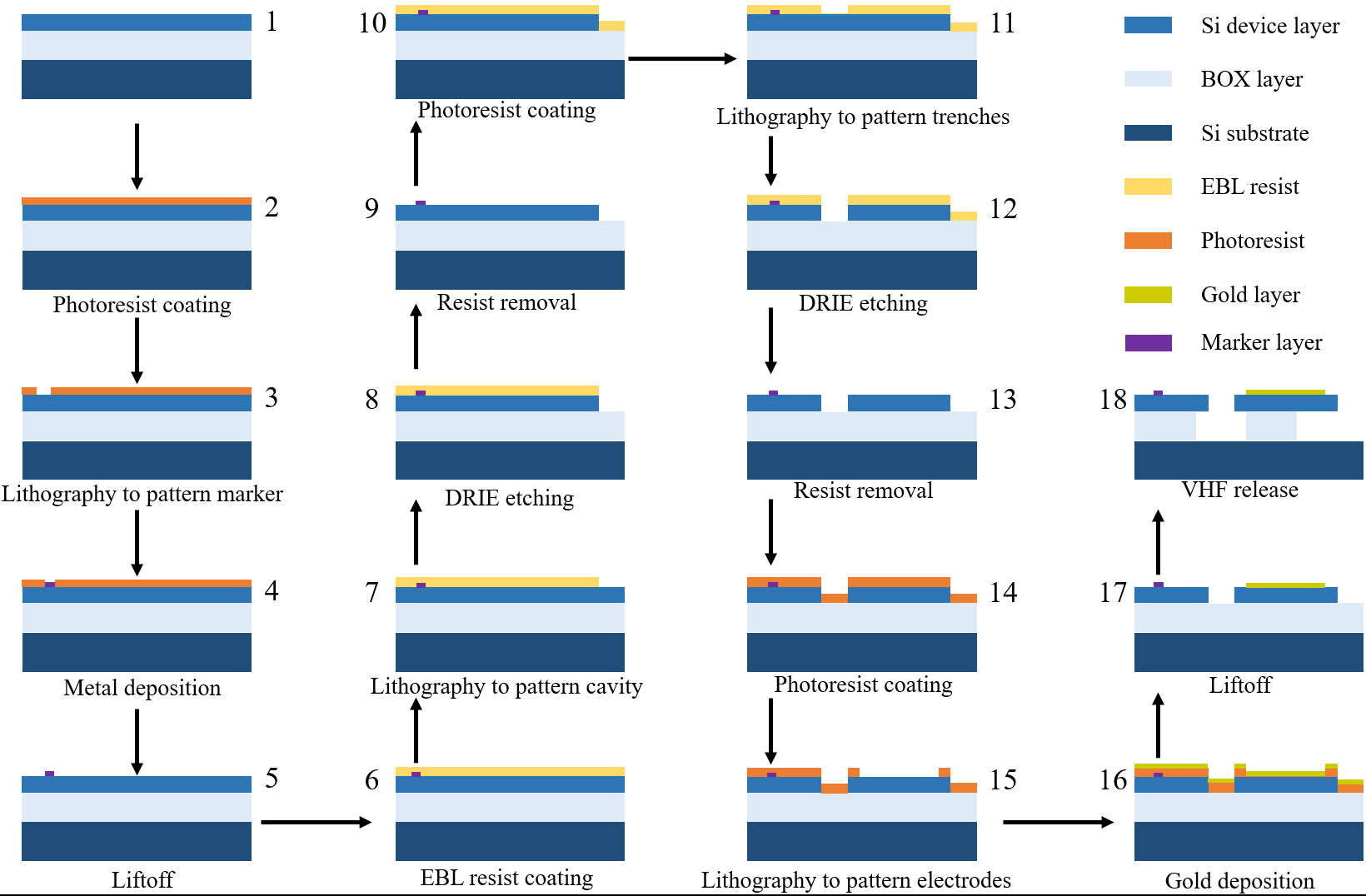


**Figure S.2.** Fabrication process for the optomechanical electrometer device.

### II. Transmission sprctrum for wavelength shfit measurement resolution

In our fiber taper evanescently coupled cavity scheme, the transmission intensity profile is a function of side-coupled open cavity and it can be given as

|  | $\text{T}\left( \text{Δ} \right)\text{=}\frac{\left\vert\text{a}_{\text{out}} \right\vert^{\text{2}}}{\left\vert\text{a}_{\text{in}} \right\vert^{\text{2}}}\text{=1}-\frac{\text{κ}_{\text{e}}}{\text{4}}\frac{\text{2κ}-\text{κ}_{\text{e}}}{\text{Δ}^{\text{2}}\text{+}{\text{κ}^{\text{2}}}/\text{4}}\text{=1}-\text{(1}-\text{T}_{\text{d}}\text{)}\frac{{\text{κ}^{\text{2}}}/\text{4}}{\text{Δ}^{\text{2}}\text{+}{\text{κ}^{\text{2}}}/\text{4}}$ | (S1) |
| --- | --- | --- |

where $\text{κ}_{\text{e}}$ is the fiber-cavity coupling rate and $\text{κ=κ}_{\text{i}}\text{+}\text{κ}_{\text{e}}$ is the total cavity decay with the intrinsic cavity damping rate $\text{κ}_{\text{i}}$. $\text{a}_{\text{in}}$and $\text{a}_{\text{out}}$ are fiber input field and output field, respectively. *T*_d_ is the dip ratio of the transmission resonance. When the optical transmission spectrum shift is used to measure the applied voltage, the variation of the applied bias voltage is determined by monitoring the shift of optical resonance. As shown in Figure S. 3a, the measured optical intensity with the laser fixed at $\Delta\text{=}-\text{κ/2}$ is obtained as,

|  | $\text{S=}\left( \text{1-}\text{T}_{\text{d}} \right)\frac{\text{Q}_{\text{o}}}{\text{ω}_{\text{c}}}\text{⋅}\text{Δω}\text{⋅}\text{P}_{\text{off}}$ | (S2) |
| --- | --- | --- |

here P_off_ is the off-resonance power intensity and $\text{Δω}$ is the resonance shift driven by the applied bias voltage. To calculate the minimum detectable resonance shift, this optical intensity at $\Delta\text{=}-\text{κ/2}$ should be larger than that of noise level P_noise_ (namely $\text{S ≥}\text{ P}_{\text{noise}}$). Therefore, the minimum detectable resonance shift is solved as

|  | $\text{R=}\frac{\text{Δω}}{\text{ω}_{\text{c}}}\text{=}\frac{\text{1}}{\text{1}-\text{T}_{\text{d}}}\text{⋅}\frac{\text{1}}{\text{Q}_{\text{o}}}\text{⋅}\frac{\text{1}}{\left( \frac{\text{P}_{\text{off }}}{\text{P}_{\text{noise }}} \right)}\text{=}\frac{\text{1}}{\text{1}-\text{T}_{\text{d}}}\text{⋅}\frac{\text{1}}{\text{Q}_{\text{o}}}\text{⋅}\frac{\text{1}}{\text{SNR}}$ | (S3) |
| --- | --- | --- |


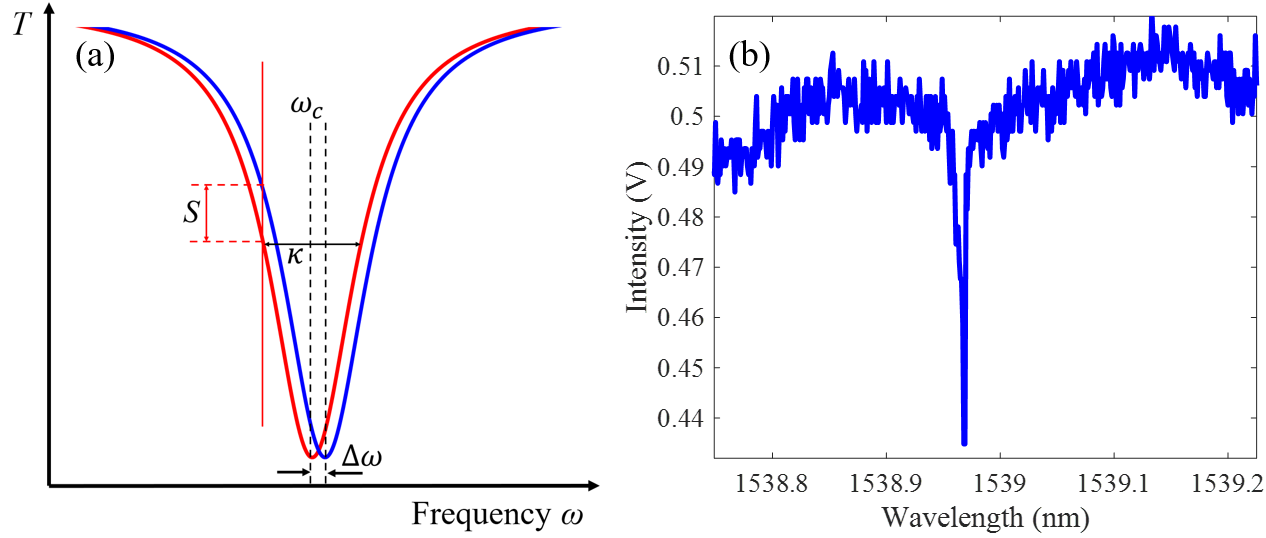


**Figure S.3.** (a) Sensing schematic of optical resonance shift for external factors measurement. (b) Measured optical resonance spectrum of TE_1,e_.

In our measurement system, the measured optical resonance in **Figure S.3b** indicates the dip ratio is T_d_= 0.83 with an estimated SNR of ~80, therefore the minimum detectable wavelength shift can be calculated as 0.75 pm at the optical resonance of 1538.96 nm with a $\text{Q}_{\text{o}}\text{ ≈ 1.5×}\text{10}^{\text{5}}$.

### III. Displacement PSD Converted from power PSD

In order to obtain the corresponding displacement noise density from the power spectral density, the relationship between the frequency-domain optical power intensity and the frequency-domain mechanical motion of the movable PCN is given as [1]:

|  | $\text{P}_{\text{m}}\text{(Ω)=}\frac{\text{dT}}{\text{d∆}}\frac{\text{Q}_{\text{o}}}{\text{ω}_{\text{c}}}\text{g}_{\text{OM}}\text{P}_{\text{det}}\text{x}\text{(Ω)}$ | (S4) |
| --- | --- | --- |

here P_det_ is the optical power received by the detector off the resonance. In our experiments, Newport 1811 balanced photodetector with transimpedance gain of g_ti_ = 40,000 V/W is used, and it corresponds to a photovoltaic conversion output of V_m_ = g_ti_P_m_. The ESA is used to obtain the power PSD of the optical signal that is modulated by the motion of mechanical resonator in the unit of V_m_^2^/Z with Z =50 Ω. Thus, the displacement PSD can be converted to power PSD intensity with dBm/Hz units by following the relation expressed as:

|  | $\text{PS}\text{D}_{\text{ESA}}\text{(Ω)=10}\text{⋅}\text{log}\left( \frac{\left( \text{g}_{\text{ti}}\text{P}_{\text{m}}\text{(Ω)} \right)^{\text{2}}}{\text{Z}}\text{⋅}\text{10}^{\text{3}} \right)$ | (S5) |
| --- | --- | --- |

With the optomechanical coupling rate determined as 13.2 GHz/nm, and T_d_ = 0.83, $\text{Q}_{\text{o}}\text{ }\text{≈}\text{ }\text{1.5×}\text{10}^{\text{5}}$, $\text{ω}_{\text{c}}\text{ = 2π×194.95 THz}$ from the experimental tests, we can obtain the displacement noise density shown in the right axis of Figure 6a in the main text, based on Eq. (S4) and (S5), which indicates the measured displacement noise floor of ~ 0.8 fm/Hz^1/2^ and a motion amplitude of ~ 210 fm/Hz^1/2^ for the fundamental mode of the mechanical resonator.

**IV. Noise analysis on the optomechanical electrometer**

Noise plays a significant role in detection sensitivity since it is the critical factor that limits sensing performance. Sources of noise in the optomechanical system are typically thermomechanical noise from the mechanical resonator, optical shot noise from the pump laser, detector noise of measurement, and backaction noise on the mechanical resonator [2,3]. Among these noise sources, optical shot noise arises as photons hit the photodetector and it behaves as white noise independent with frequency. Similarly, the detection noise caused by the dark current is defined by its noise-equivalent-power (NEP, for Newport 1811, $\text{NEP= 2.5 }\text{pW}\text{/}\sqrt{\text{Hz}}$). Note that, this RF readout method is different from the optical transmission intensity measurement in which both the optical shot noise and detector noise contribute significantly to the fundamental limit of detection. Thus, in this work, only the thermomechanical noise and backaction noise are taken into account to evaluate our measured detection noise floor.

1. **Thermomechanical noise**

In this optomechanical electrometer, the working mechanism of this sensor is based on the electrostatic force-induced perturbation on the motion of the movable PCN resonator. Displacement of the mechanical resonator is closely related to applied force by the mechanical susceptibility χ_xx_ as defined in Fourier transform as [4],

|  | $\text{x(Ω)=}\text{χ}_{\text{xx}}\text{(Ω)}\left[ \text{F}_{\text{es}}\text{(Ω)+}\text{F}_{\text{N}}\text{(Ω)} \right]$ | (S6) |
| --- | --- | --- |

where, $\text{F}_{\text{es}}\text{(Ω)}$ and $\text{F}_{\text{N}}\text{(Ω)}$ are the applied electrostatic force and noise force mainly arising from thermal Brownian motion, respectively. The thermomechanical noise spectrum is generally used to analyze the thermal Brownian motion by the following displacement PSD [5].

|  | $\text{S}_{\text{xx}}^{\text{th}}\text{(Ω)=}\frac{\text{4}\text{k}_{\text{B}}\text{T}_{\text{e}}\text{Ω}_{\text{m}}}{\text{Q}_{\text{m}}}\frac{\text{1}}{\text{m}_{\text{eff}}\left[ \left( \text{Ω}^{\text{2}}-\text{Ω}_{\text{m}}^{\text{2}} \right)^{\text{2}}\text{+}\left( \frac{\text{Ω}\text{Ω}_{\text{m}}}{\text{Q}_{\text{m}}} \right)^{\text{2}} \right]}$ | (S7) |
| --- | --- | --- |

where k_B_ is Boltzmann’s constant, and T_e_ is operation temperature (300 K for our experimental environment). Thermomechanical noise is frequency-independent in the displacement PSD spectrum. Besides, the displacement PSD of the mechanical resonator is greatly enhanced at the resonance of mechanical mode ($\text{Ω}$ = $\text{Ω}$_m_) due to the mechanical susceptibility $\text{χ}_{\text{xx}}$.

**(B) Backaction noise**

The momentum of light exerts random radiation pressure on the mechanical resonator, which causes the backaction noise. The backaction noise radiation pressure is similar to the white noise for frequencies of the relevance of the mechanical resonance [5]. Optical noise arising from quantum backaction noise can be expressed by the force PSD spectrum as,

|  | $\text{S}_{\text{FF}}^{\text{BA}}\text{(Ω, Δ)=2}\left( \text{2ħ}\text{g}_{\text{OM}} \right)^{\text{2}}\frac{\text{n}_{\text{c}}\text{(Δ)}}{\text{κ}}$ | (S8) |
| --- | --- | --- |

where *n_c_* is the cavity photon number and it can be calculated as

|  | $\text{n}_{\text{c}}\text{(Δ)=}\frac{\text{κ}_{\text{e}}}{\text{2}}\frac{\text{1}}{\text{Δ}^{\text{2}}\text{+}\left( \frac{\text{κ}}{\text{2}} \right)^{\text{2}}}\frac{\text{P}_{\text{in}}}{\text{ħ}\text{ω}_{\text{l}}}$ | (S9) |
| --- | --- | --- |

At the operation wavelength of $\text{Δ=}\frac{\text{κ}}{\text{2}}$, the backaction noise can be rewritten as

|  | $\text{S}_{\text{FF}}^{\text{BA}}\text{(Ω)=}\frac{\text{8ħ}\text{g}_{\text{OM}}^{\text{2}}\text{Q}_{\text{o}}^{\text{2}}\left( \text{1}-\sqrt{\text{T}_{\text{d}}} \right)\text{P}_{\text{in}}}{\text{ω}_{\text{c}}^{\text{2}}\text{ω}_{\text{l}}}$ | (S10) |
| --- | --- | --- |

Converting the force noise $\text{S}_{\text{FF}}^{\text{BA}}\text{(Ω)}$ to the frequency-dependent displacement noise $\text{S}_{\text{xx}}^{\text{BA}}\text{(Ω)}$,

|  | $\text{S}_{\text{xx}}^{\text{BA}}\text{(Ω)=2}\left( \frac{\text{2ħ}\text{g}_{\text{OM}}}{\text{m}} \right)^{\text{2}}\frac{\text{n}_{\text{c}}}{\text{κ}}\left\vert\text{χ}_{\text{xx}}\text{(Ω)} \right\vert^{\text{2}}$ | (S11) |
| --- | --- | --- |

Therefore, the backaction noise at the mechanical resonance can be given from Eq.(S10) and Eq.(S11),

|  | $\text{S}_{\text{xx}}^{\text{BA}}\text{(Ω)=2}\left( \frac{\text{2ħ}\text{g}_{\text{OM}}\text{Q}_{\text{m}}}{\text{m}\text{Ω}_{\text{m}}^{\text{2}}} \right)^{\text{2}}\frac{\left( \text{1}-\sqrt{\text{T}_{\text{d}}} \right)}{\text{κ}}\frac{\text{P}_{\text{in}}}{\text{ℏ}\text{ω}_{\text{c}}}$ | (S12) |
| --- | --- | --- |

By setting the input power as 30 μW for optomechanical oscillation under the threshold power, the photon number in the cavity $\text{n}_{\text{c}}\approx$410, $\text{Ω}_{\text{m}}\text{/2π= 1.286 MHz}$, $\text{Q}_{\text{m}}\text{≈1280}$, m=22.6 pg, Q_o_ $\approx\text{1.0×}\text{10}^{\text{5}}$, $\text{λ}_{\text{c}}$=1538 nm, $\text{g}_{\text{OM}}\text{/2π ≈ 13.2 GHz/nm}$, the calculated noise existed in the optomechanical system across the frequency range is shown in **Figure S4**. It can be seen that the thermomechanical noise dominates the total noise of the optomechanical system, and hence we take the thermal noise floor in Fig. 5a in the main text to represent the calculated noise level.

**Figure S.4.** Numerical displacement noise floor of the optomechanical electrometer at room temperature and low-pressure environment.

**V. Self-sustained optomechanical oscillation**

When the pump light intensity is gradually increased, the damping oscillation of the mechanical resonator turns into a driven oscillation state in which its resonant mechanical linewidth could approach approximately infinitesimal. This phenomenon is also named self-sustained optomechanical oscillation [6,7], and it can be represented in the form of optical damping of the optomechanical system as below

|  | $\text{Γ}_{\text{opt}}\text{=}\text{g}_{\text{0}}^{\text{2}}n_{\text{c}}\left( \frac{\text{κ}}{\left( \text{Δ+}\text{Ω}_{\text{m}} \right)^{\text{2}}\text{+(κ/2}\text{)}^{\text{2}}}-\frac{\text{κ}}{\left( \text{Δ}-\text{Ω}_{\text{m}} \right)^{\text{2}}\text{+(κ/2}\text{)}^{\text{2}}} \right)$ | (S13) |
| --- | --- | --- |

where $\text{g}_{\text{0}}\text{=}\text{g}_{\text{OM}}\sqrt{\text{ℏ/(2m}\text{Ω}_{\text{m}}\text{)}}$ is the optomechanical single-photon coupling strength. The yielding the full effective mechanical damping rate can be written as $\text{Γ}_{\text{eff}}\text{=}\text{Γ}_{\text{opt}}\text{+}\text{Γ}_{\text{m}}$. Because $\text{Γ}_{\text{opt}}$ can be both positive and negative, it can either increase or decrease the mechanical damping rate, i.e., cause extra damping or anti-damping. In an optomechanical system with a sufficiently strong blue-detuned laser drive, the mechanical oscillations can display anti-damping as $\text{Γ}_{\text{opt}}\text{<0}$. In particular, assuming effective mechanical damping rate approaches close to zero,

|  | $\text{Γ}_{\text{eff}}\text{=}\text{Γ}_{\text{opt}}\text{+}\text{Γ}_{\text{m}}\text{=0}$ | (S14) |
| --- | --- | --- |

Therefore, the threshold pump power for the self-sustained optomechanical oscillation can be solved as,

|  | $\text{P}_{\text{th}}\text{=}\frac{\text{Ω}_{\text{m}}}{\text{Q}_{\text{m}}}\frac{\text{ħ}\text{ω}_{\text{l}}\left( \text{Δ}^{\text{2}}\text{+(κ/2}\text{)}^{\text{2}} \right)}{\text{g}_{\text{0}}^{\text{2}}\text{κ}_{\text{ex}}\text{κ}}\left( \frac{\text{κ}}{\left( \text{Δ+}\text{Ω}_{\text{m}} \right)^{\text{2}}\text{+(κ/2}\text{)}^{\text{2}}}-\frac{\text{κ}}{\left( \text{Δ}-\text{Ω}_{\text{m}} \right)^{\text{2}}\text{+(κ/2}\text{)}^{\text{2}}} \right)^{\text{-1}}$ | (S15) |
| --- | --- | --- |

In the unresolved sideband ($\text{Ω}_{\text{m}}\text{<<}\text{κ}$) of our experimental system, Eq.(S15) can be simplified as,

|  | $\text{P}_{\text{th}}\text{=}\frac{\text{ħ}\text{ω}_{\text{l}}}{\text{4}\text{g}_{\text{0}}^{\text{2}}\text{κ}_{\text{ex}}\text{κ}\text{Q}_{\text{m}}}\frac{\text{Δ}^{\text{2}}\text{+(κ/2}\text{)}^{\text{2}}}{\text{Δ}}$ | (S16) |
| --- | --- | --- |

According to Eq. (S16), when the laser-detuning is set as $\text{Δ=κ}\text{/(}\text{2}\sqrt{\text{5}}\text{)}$, the minimal threshold power can be obtained to achieve the self-sustained optomechanical oscillation,

|  | $\text{P}_{\text{th,min}}\text{=}\frac{\text{27}}{\text{400}\sqrt{\text{5}}}\frac{\text{ħ}\text{ω}_{\text{l}}}{\text{g}_{\text{0}}^{\text{2}}}\frac{\text{κ}^{\text{4}}}{\text{Ω}_{\text{m}}\text{κ}_{\text{ex}}}$ | (S17) |
| --- | --- | --- |

Based on the experimental results from our optomechanical measurement, the minimal threshold power is calculated as about 138 μW.

**VI. Technical performance of electrometers**

**Table 1.** Performance comparison of various micro- and nano-electrometry techniques

| **Year [Ref]** | **Sensor type** | **Transduction mechanism** | **Material** | **Temperature** | **Pressure** | **Resolution** |
| --- | --- | --- | --- | --- | --- | --- |
| 2008 [8] | SET | Tunneling and reflectometry | Silicon | 10 mK | N.A. | $\text{1.0×}\text{10}^{\text{-5}}\text{ e}\text{Hz}^{\text{-1/2}}\text{ @15MHz}$ |
| 2012 [9] | SET | Tunneling and reflectometry | Si/SiGe | 0.3 K | N.A. | $\text{4.0×}\text{10}^{\text{-6}}\text{ e}\text{Hz}^{\text{-1/2}}\text{ @1kHz}$ |
| 2008 [10] | MEMS vibrating reed | Electrostatic actuation and probing | SOI | 300 K | Ambient | $\text{6}\text{ e}\text{Hz}^{\text{-1/2}}\text{ @5.7kHz}$ |
| 2013 [11] | MEMS vibrating reed | Electrostatic actuation and probing | SOI | 300 K | Ambient | $\text{23}\text{ e}\text{Hz}^{\text{-1/2}}$ |
| 2018 [12] | MEMS vibrating reed | Electrostatic actuation and probing | SOG | 300 K | Ambient | $\text{1.03}\text{ e}\text{Hz}^{\text{-1/2}}\text{ @5.7kHz}$ |
| 2016 [13] | MEMS coupled resonator | Electrostatic actuation and probing | SOI | 300 K | 20 mTorr | 1.269 fC |
| 2015 [14] | MEMS resonator | Mechanical oscillation and probing | SOI | 300 K | 40 mTorr | 21 fC under 4 ppm frequency fluctuation |
| 2017 [15] | MEMS resonator | Mechanical oscillation and probing | SOI | 300 K | 37 mTorr | 20300$\text{ e}\text{Hz}^{\text{-1/2}}$ |
| 2018 [16] | MEMS resonator | Mechanical oscillation and probing | SOI | 300 K | 22 mTorr | 2.6 fC under 0.46 ppm frequency fluctuation. |
| 2020 [17] | MEMS resonator | Mechanical oscillation and probing | SOI | 300 K | 20 mTorr | 0.17 $\text{e}\text{Hz}^{\text{-1/2}}$ |
| This work | Optomechanical cavity | Optomechanical oscillation and RF readout | SOI | 300 K | 3.7 mTorr | $\text{1.33}\text{×}\text{10}^{\text{-2}}\text{ e}\text{Hz}^{\text{-1/2}}$ |

**Reference**

1. Safavi-Naeini, A.H.; Chan, J.; Hill, J.T.; Gröblacher, S.; Miao, H.; Chen, Y.; Aspelmeyer, M.; Painter, O. Laser noise in cavity-optomechanical cooling and thermometry. *New Journal of Physics* **2013**, *15*, 035007.

2. Wu, M.; Zeuthen, E.; Balram, K.C.; Srinivasan, K. Microwave-to-optical transduction using a mechanical supermode for coupling piezoelectric and optomechanical resonators. *Physical Review Applied* **2020**, *13*, 014027.

3. Kaviani, H.; Ghobadi, R.; Behera, B.; Wu, M.; Hryciw, A.; Vo, S.; Fattal, D.; Barclay, P. Optomechanical detection of light with orbital angular momentum. *Optics express* **2020**, *28*, 15482-15496.

4. Aspelmeyer, M.; Kippenberg, T.J.; Marquardt, F. Cavity optomechanics. *Reviews of Modern Physics* **2014**, *86*, 1391.

5. Krause, A.G.; Winger, M.; Blasius, T.D.; Lin, Q.; Painter, O. A high-resolution microchip optomechanical accelerometer. *Nature Photonics* **2012**, *6*, 768-772.

6. Huang, J.; Li, Y.; Chin, L.K.; Cai, H.; Gu, Y.; Karim, M.F.; Wu, J.; Chen, T.; Yang, Z.; Hao, Y. A dissipative self-sustained optomechanical resonator on a silicon chip. *Applied Physics Letters* **2018**, *112*, 051104.

7. Guha, B.; Allain, P.E.; Lemaitre, A.; Leo, G.; Favero, I. Force sensing with an optomechanical self-oscillator. *Physical Review Applied* **2020**, *14*, 024079.

8. Angus, S.; Ferguson, A.; Dzurak, A.; Clark, R. A silicon radio-frequency single electron transistor. *Applied Physics Letters* **2008**, *92*, 112103.

9. Yuan, M.; Yang, Z.; Savage, D.; Lagally, M.; Eriksson, M.; Rimberg, A. Charge sensing in a si/sige quantum dot with a radio frequency superconducting single-electron transistor. *Applied Physics Letters* **2012**, *101*, 142103.

10. Lee, J.; Zhu, Y.; Seshia, A. Room temperature electrometry with sub-10 electron charge resolution. *Journal of Micromechanics and Microengineering* **2008**, *18*, 025033.

11. Jaramillo, G.; Buffa, C.; Li, M.; Brechtel, F.J.; Langfelder, G.; Horsley, D.A. Mems electrometer with femtoampere resolution for aerosol particulate measurements. *IEEE Sensors Journal* **2013**, *13*, 2993-3000.

12. Jalil, J.; Ruan, Y.; Zhu, Y. Room-temperature sensing of single electrons using vibrating-reed electrometer in silicon-on-glass technology. *IEEE Electron Device Letters* **2018**, *39*, 1928-1931.

13. Zhang, H.; Li, B.; Yuan, W.; Kraft, M.; Chang, H. An acceleration sensing method based on the mode localization of weakly coupled resonators. *Journal of microelectromechanical systems* **2016**, *25*, 286-296.

14. Zhao, J.; Ding, H.; Xie, J. Electrostatic charge sensor based on a micromachined resonator with dual micro-levers. *Applied Physics Letters* **2015**, *106*, 233505.

15. Chen, D.; Zhao, J.; Wang, Y.; Xie, J. An electrostatic charge sensor based on micro resonator with sensing scheme of effective stiffness perturbation. *Journal of Micromechanics and Microengineering* **2017**, *27*, 065002.

16. Chen, D.; Zhao, J.; Wang, Y.; Xu, Z.; Xie, J. Sensitivity manipulation on micro-machined resonant electrometer toward high resolution and large dynamic range. *Applied Physics Letters* **2018**, *112*, 013502.

17. Chen, D.; Zhang, H.; Sun, J.; Pandit, M.; Sobreviela, G.; Wang, Y.; Zhang, Q.; Chen, X.; Seshia, A.; Xie, J. Ultrasensitive resonant electrometry utilizing micromechanical oscillators. *Physical Review Applied* **2020**, *14*, 014001.
